# The evolution of nuclear auxin signalling

**DOI:** 10.1186/1471-2148-9-126

**Published:** 2009-06-03

**Authors:** Ivan A Paponov, William Teale, Daniel Lang, Martina Paponov, Ralf Reski, Stefan A Rensing, Klaus Palme

**Affiliations:** 1 Botany, Faculty of Biology, University of Freiburg, Schänzlestrasse 1, 79104 Freiburg, Germany; 2 Plant Biotechnology, Faculty of Biology, University of Freiburg, Schänzlestrasse 1, 79104 Freiburg, Germany; 3 FRISYS, Faculty of Biology, University of Freiburg, Schänzlestrasse 1, 79104 Freiburg, Germany

## Abstract

**Background:**

The plant hormone auxin directs many aspects of plant growth and development. To understand the evolution of auxin signalling, we compared the genes encoding two families of crucial transcriptional regulators, *AUXIN RESPONSE FACTOR *(*ARF*) and *AUXIN/INDOLE-3-ACETIC ACID *(*Aux/IAA*), among flowering plants and two non-seed plants, *Physcomitrella patens *and *Selaginella moellendorffii*.

**Results:**

Comparative analysis of the *P. patens, S. moellendorffii *and *Arabidopsis thaliana *genomes suggests that the well-established rapid transcriptional response to auxin of flowering plants, evolved in vascular plants after their divergence from the last common ancestor shared with mosses. An N-terminally truncated ARF transcriptional activator is encoded by the genomes of *P. patens *and *S. moellendorffii*, and suggests a supplementary mechanism of nuclear auxin signalling, absent in flowering plants. Site-specific analyses of positive Darwinian selection revealed relatively high rates of synonymous substitution in the *A. thaliana *ARFs of classes IIa (and their closest orthologous genes in poplar) and Ib, suggesting that neofunctionalization in important functional regions has driven the evolution of auxin signalling in flowering plants. Primary auxin responsive gene families (GH3, SAUR, LBD) show different phylogenetic profiles in *P. patens*, *S. moellendorffii *and flowering plants, highlighting genes for further study.

**Conclusion:**

The genome of *P. patens *encodes all of the basic components necessary for a rapid auxin response. The spatial separation of the Q-rich activator domain and DNA-binding domain suggests an alternative mechanism of transcriptional control in *P. patens *distinct from the mechanism seen in flowering plants. Significantly, the genome of *S. moellendorffii *is predicted to encode proteins suitable for both methods of regulation.

## Background

The evolution of signal transduction pathways since the divergence of plants and animals has been influenced by very different selection pressures. Hormone signalling, though analogous in both kingdoms, differs in the signalling molecules employed as well as in their perception and mode of action. Plants are adapted to a sessile lifestyle, being able continuously to form new organs during their postembryonic development. This process, in addition to embryonic development, is closely associated with specific growth regulators, effective at low concentrations. The signalling pathways of these growth regulators (also known as phytohormones) are relatively well understood, but their evolution, as well as their relationship to the evolution of embryonic and post-embryonic development in the plant kingdom, is less clear [1].

Auxin, one such phytohormone, is a principal regulator of growth and development in flowering plants [2], quickly triggering the transcription of auxin-responsive genes [3,4]. Proteins of two related families, AUXIN RESPONSIVE FACTOR (ARF) and AUXIN/INDOLE-3-ACETIC ACID (Aux/IAA), act together to regulate this transcription [5,6]. In flowering plants, ARF proteins possess a conserved DNA-binding domain which recognizes auxin responsive elements (AuxREs): short motifs which are found in the promoter sequences of many auxin-responsive genes [7,8]. Most ARFs, and all Aux/IAAs also contain a conserved dimerization domain which mediates protein-protein interactions within and between both protein families [9,10]. The middle region which joins ARF DNA-binding and dimerization domains is highly divergent and may be glutamine (Q) rich [11]. Those ARFs which contain such Q-rich regions are thought to be activators of gene transcription [11,12]. Conversely, those ARFs which repress gene transcription lack glutamine (or in one case methionine) -rich regions.

The N-terminal region of Aux/IAA proteins contains two other domains: domain I and II. Domain I contains a short amphiphilic repression motif, which binds to the co-repressor TOPLESS, enabling Aux/IAAs to repress ARF function [13,14]. Domain II contains a degron: a motif sufficient to signal Aux/IAAs for proteasome-mediated degradation [6,15,16]. Specific point mutations in domain II confer strong, auxin insensitive phenotypes [17].

At low cellular auxin concentrations, Aux/IAA proteins dimerize with ARF transcriptional activators, repressing their activity [18]. Auxin itself can bind at the interface of Aux/IAA proteins and TIR1-family F-box proteins, components of specific SCF E3 ubiquitin ligases, directly promoting their interaction. Accordingly, at high cellular auxin concentrations, Aux/IAAs are ubiquitinated and subsequently degraded [19-21]. Degradation of Aux/IAA proteins then allows ARF-mediated, auxin-dependent gene transcription.

*Physcomitrella patens *(a moss), *Selaginella moellendorffii *(a vascular non-seed plant) and angiosperms diverged from each other at between 700 and 450 million years ago [22]. The genomes of both *P. patens *and *S. moellendorffii *encode all the proteins necessary for this primary auxin response [23,24]. Furthermore, *P. patens *has been shown to both synthesize auxin, and respond to exogenously applied auxin [25,26]. Here we use the complete genomic sequences of *P. patens *and *S. moellendorffii *to address how a relatively simple signalling mechanism has evolved into, in flowering plants, a central regulator of many essential and diverse developmental processes. A driving force of this evolution has been positive Darwinian selection. Such positive selection is a measure of the adaptation of amino acid sequences following a gene duplication event. The unambiguous indicator of positive selection, a high ratio of non-synonymous (dN) to synonymous (dS) nucleotide substitutions, was detected in the flowering plant ARFs.

Based on a comparative analysis of the fully-sequenced genomes of *P. patens*, *S. moellendorffii*, and selected flowering plants, we are able to draw conclusions about ancestral auxin target genes and signalling mechanisms, and about the pressures which have driven the radiation of auxin-signalling genes in flowering plants.

## Results and discussion

Endogenous auxin is a widely used signalling molecule in vascular plants, but is also found in bryophytes, algae and prokaryotes [25]. In the present study, we identify similarities and differences between the auxin signalling components in moss and flowering plants by comparing the fully sequenced genomes of *P. patens *with those of model flowering plant species. Additional support, where appropriate, is drawn from the genome of *S. moellendorffii*, a vascular non-seed plant. Here we present an analysis of two gene families central to auxin signalling: the *ARFs*, encoding transcription factors, and *Aux/IAAs*, encoding their repressors. We also analyze three families of primary auxin responsive genes, which are among the first targets of auxin-induced transcription in flowering plants.

### Aux/IAA

**Figure 1:**
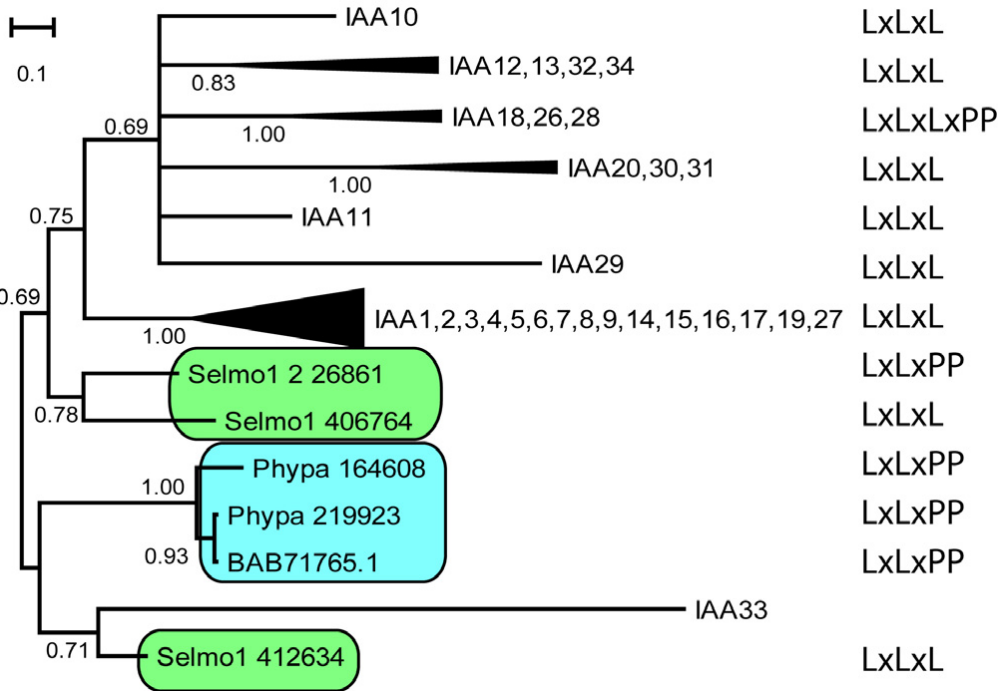
**Phylogenetic relationship of *A. thaliana*, *S. moellendorffii *and *P. patens *Aux/IAA proteins (Bayesian inference)**. The presence of the domain I motif is marked.

The *P. patens *genome encodes three Aux/IAA proteins (PpAux/IAA) (Figure 1). These proteins, at between 484 and 503 amino acids, are significantly longer than all 29 *A. thaliana *Aux/IAA (AtAux/IAA) family members (the longest of which, IAA9, consists of 338 amino acids). The three Aux/IAAs of *S. moellendorffii *vary in length between 170 and 421 amino acids. Aux/IAA proteins typically comprise four domains: domain I confers the proteins' transcriptional repressor function, domain II is a degradation motif, and domains III and IV form a protein dimerization domain, evolutionarily related to the C-terminal dimerization (CTD) domain of ARF proteins.

#### Domain I

In all but one *Arabidopsis *Aux/IAA protein, domain I contains a sequence of amino acids reminiscent of an ERF-associated amphiphilic repression (EAR) motif. This LxLxL domain I motif interacts directly with TOPLESS (TPL), a transcriptional co-repressor. This interaction leads to a repression of the ARF-dependent transcription of a reporter gene driven by the DR5 promoter, a synthetic auxin-sensitive marker containing repeated TGTCTC auxin response elements (AuxREs) [13,14].

PpAux/IAAs do not contain an LxLxL motif in domain I. Instead they all contain a similar LxLxPP motif (Figure 1, Additional file 1). A corresponding and overlapping LxLxLxPP motif was found in three AtAux/IAAs (IAA18, 26 and 28), forming a cluster with good bootstrap support (Figure 1, Additional file 1). The genome of *S. moellendorffii *encodes three Aux/IAA proteins, one of which contains an LxLxPP motif in domain I. The other two contain the LxLxL motif typical of flowering plants (Figure 1, Additional file 1). The genes containing LxLxL motifs found in domain I of *S. moellendorffii *and flowering plants do not form monophyletic groups. Therefore the motif is likely to have become established at least twice in each lineage.

Of the 35 Aux/IAAs encoded by the *Populus trichocarpa *genome, 27 are predicted to contain an LxLxL motif and six an LxLxPP motif [27]. In rice, it is predicted that 27 out of 33 Aux/IAAs contain an LxLxL motif; of these, two contain an LxLxLxPP motif. In rice, one Aux/IAA contains LxLxPP (Table 1). At present, there is no evidence that this LxLxPP motif, in any species, represents a functional repression domain. There are also no experimental data available which test the role of flowering plant Aux/IAAs which contain no apparent functional domain I motif. These proteins may function as competitive regulators of the auxin response. Gaining empirical functional evidence on these proteins will allow hypotheses on their evolution to be tested.

**Table 1:** Number of ARFs and Aux/IAA with different domains and motifs.

Proteins	*P.p*.	*S.m*.	*A.t*.	*P.t*. *	*O.s*.	*S.b*.	*V.v*.	*G.m*.
ARF with DBD domain								
Total number	12	7	23	38 (39)	25	25	20	59
No III &IV domain	1	1	4	12 (6)	6	6	5	12
Q-rich	0	3	4**	8 (10)	8	8	3	18
								
ARF without DBD domain								
Only III&IV domain	1	4	0	11 (0)	0	0	0	8
Q-rich, no DBD domain	2	2	0	0 (0)	0	0	0	0
								
Aux/IAA								
Total number	3	3	29	33 (35)	33	26	27	62
LxLxL	0	2	28	23 (31)	27	21	17	55
LxLxPP	3	1	3	0 (6)	3	3	2	4
No domain II	0	0	5	1 (2)	6	2	8	21
No KR domain	0	0	10	9 (10)	11	7	9	22

(P.p. – P. patens, S.m. – S. moellendorffii, A.t. – A. thaliana, P.t. – P. trichocarpa, O.s. – O. sativa, S.b. – S. bicolor, V.v. – Vitis vinifera G.m – G. max).

* The number of ARFs and Aux/IAAs from Kalluri et al. [27] is shown in brackets.

** In addition, one *A. thaliana *ARF, ARF5, has a methionine-rich middle region.

There is one homologous position for the EAR-like motif of domain I. Based on the alignment of *A. thaliana*, *S. moellendorffii *and *P. patens *Aux/IAAs (Additional file 1) this domain I motif can be expanded to LXL [A, G] [L, P] [P, G, S, T]. This allows the detection of domain I in all sequences tested of these three species. If expanded further, an [L, I]X [L, I] [A, G] [L, P] [P, G, S, T] motif can, according to our present knowledge, be used to detect domain I in all land plant Aux/IAAs. This analysis does not preclude the possibility that other non-homologous domains serving a similar function are also present.

Mutations in the leucine positions of the domain I motif of *Arabidopsis *have been shown to result in significantly weaker repression of ARF-mediated transcription to Aux/IAA proteins [14]. Nevertheless, the widespread conservation of the LxLxPP sequence suggests it is a functional motif. The predicted presence in *P. patens *of two TPL-like transcriptional co-repressors (Additional file 2) also suggests the LxLxPP motif is able to inhibit (at least to some extent) ARF-mediated transcription. In flowering plants, however, the LxLxPP sequence appears to have been superseded by the LxLxL domain (Additional file 1). Notably, the genome of *S. moellendorffii *encodes proteins predicted to contain both motifs. Although the relative efficiency of domain I-dependent transcriptional repression in non-seed plants (via the LxLxPP motif) and flowering plants (via the LxLxL motif) is not possible to assess with the data that are currently available, it is highly significant, as they would be expected profoundly to influence the role of auxin-dependent transcriptional activation.

#### Domain II

The alignment of domain II from several Aux/IAA proteins indicates that not all 13 amino acids of the consensus sequence, which in flowering plants mediate the specific proteasomal degradation of Aux/IAAs in response to auxin, are faithfully conserved (Additional file 3). Nevertheless, a central core of five residues, representing amino acids 4–8 (GWPPV), is required for targeted protein degradation [16]. Though not sufficient to confer protein instability to a luciferase reporter fusion on its own, the functionally essential central motif (which can be represented by VGWPP [L, V, I]) is conserved in all Aux/IAAs, including those from *P. patens *and *S. moellendorffii *(Additional file 3).

Aux/IAAs are degraded after domain II binds to the TIR1 family of F-box proteins [19-21]. The presence of the core motif of domain II and four paralogs of the Aux/IAA-specific TIR1 family of F-box proteins (Additional file 4) in *P. patens*, suggests that PpAux/IAAs are degraded in an auxin-dependent manner. Homology modelling has shown that the auxin binding pocket of PpTIR1 is intact [26]. Together, these data suggest that auxin-mediated targeted protein degradation is relevant in *P. patens*, and that the relatively slow response of *P. patens *to auxin [28,29] is not due to an impaired ability to degrade Aux/IAAs in response to auxin.

#### Diversification of Aux/IAA

The dramatic radiation of *Aux/IAA *genes in land plants (from three in *P. patens *to 29 in *A. thaliana*, 35 in *P. trichocarpa *and 33 *O. sativa*) (Figure 1, Table 1) underpins a corresponding increase in the complexity of auxin signalling. After the separation of lycophytes and seed plants, the *Aux/IAA *family in the *A. thaliana *lineage was expanded by 25 additional duplication events (Additional file 5). To test whether this radiation has been driven by neofunctionalization at the amino acid level (for example in response to specific changes in ARF protein structures), rates of positive selection were measured [30]. Specifically, we applied a likelihood ratio test (LRT) to selected *Aux/IAA *sub-families of *A. thaliana *and *P. trichocarpa*, and compared data fits to two models: M1 vs. M2 and M7 vs. M8 (Table 2; Additional file 6, A). A comparison of these models measures the likelihood that differences in non-synonomous/synonomous substitution ratios happened by chance. For Aux/IAA proteins, no significant differences between test and null hypotheses were found in any of the data sets tested.

**Table 2:** Sites under PDS in the *A. thaliana*/*P. trichocarpa *Aux/IAA gene family: „Site-specific analysis".

Site-specific analyses	n	dN/dS (ω) under M0	2Δℓ M2 vs. M1 (df 2)	2Δℓ M8 vs. M7 (df 2)	Parameter estimates under M8	Positively selected sites under M2 (BEB)	Positively selected sites under M8 (BEB)
**Aux/IAA**							
Node IAA1	9	0.112	0.00	0.00	ρ_0 _= 1.000 (ρ_1 _= 0.000) (ρ = 0.285) q = 1.705 ω = 2.40	None	22
Node IAA5	5	0.132	0.00	1.44	ρ_0 _= 0.944 (ρ_1 _= 0.056) (ρ = 0.315) q = 1.634 ω = 2.11	None	21 23 39 40 68
Node IAA7	10	0.090	0.00	4.62	ρ_0 _= 0.948 (ρ_1 _= 0.052) (ρ = 0.318) q = 3.848 ω = 1.00	None	16 19 40 42 49 75 80 81
Node IAA8	7	0.125	0.00	0.00	ρ_0 _= 1.000 (ρ_1 _= 0.000) (ρ = 0.537) q = 2.898 ω = 2.302	None	10 11 171
Node IAA10	6	0.076	0.00	0.10	ρ_0 _= 0.977 (ρ_1 _= 0.023) (ρ = 0.522) q = 3.630 ω = 1.000	None	124
Node IAA18	6	0.112	0.00	0.00	ρ_0 _= 1.000 (ρ_1 _= 0.000) (ρ = 0.505) q = 0.301 ω = 5.133	None	20 115 116
Node IAA20	5	0.155	0.74	4.54	ρ_0 _= 0.898 (ρ_1 _= 0.102) (ρ = 0.617) q = 4.304 ω = 1.307	43	13 43 49

Each comparison has n sequences, dN/dS is average ratio over sites under a codon model with one ω. Bold underline, PP ≥ 0.99 of being under positive selection; bold, 0.99>PP ≥ 0.95; italic, 0.95>PP ≥ 0.90; underline, 0.90>PP ≥ 0.70; normal, 0.70>PP > 0.50

*Aux/IAA *genes have been retained in the *A. thaliana *genome at a high rate. A two-way analysis of variance (ANOVA) test of microarray data has previously shown that the gene expression patterns of Aux/IAA sister pairs of *A. thaliana *are significantly different [31]. We extended this analysis by widening the conditions tested. Two-way ANOVA results for ten pairs of *Aux/IAA *genes are reported as graphs of expression levels at 63 conditions in Additional file 7, A–J (after [32]). All ten sister pairs of *Aux/IAA *showed significant gene (G), sample (S), and gene by sample (GxS) effects (Additional file 7, A–J).

*Aux/IAA *genes have radiated through segmental duplication events [33]. In *P. trichocarpa *and *O. sativa*, both *ARF *and *Aux/IAA *gene families have been expanded, also largely due to segmental duplication [27,34,35]. After such events, the gradual appearance of deleterious mutations generally leads to the loss of one of the duplicated genes [36]. If both gene copies are retained, there is a higher probability that mutations leading to a split in the expression pattern of the ancestral gene between duplicated genes, rather than mutations that lead to a new function in one copy, have occurred [37]. Such a split can occur through changes in transcription-factor binding sites within promoter regions that result in differential expression of the two gene copies. We therefore conclude that changes in expression pattern have driven *Aux/IAA *radiation. Indeed, when compared to amino acid substitution rates, changes in expression pattern contribute more to Aux/IAA function [38,39]. Studies in *P. trichocarpa *[27] also showed that genes of the expanded PtIAA3 subgroup, which is represented by six members, are differentially transcribed. These data lend further support to the hypothesis that the diversification of Aux/IAA family members in flowering plants has been sustained by changes in their expression patterns.

### ARF

In *A. thaliana*, all ARFs contain a DNA binding domain, but some lack a C-terminal dimerization domain (CTD). The genomes of *S. moellendorffii *and *P. patens *also encode ARFs with C-terminal truncations, as well as those with N-terminal truncations. All of these variants are discussed below.

#### Full-length and C-terminally truncated ARFs

**Figure 2:**
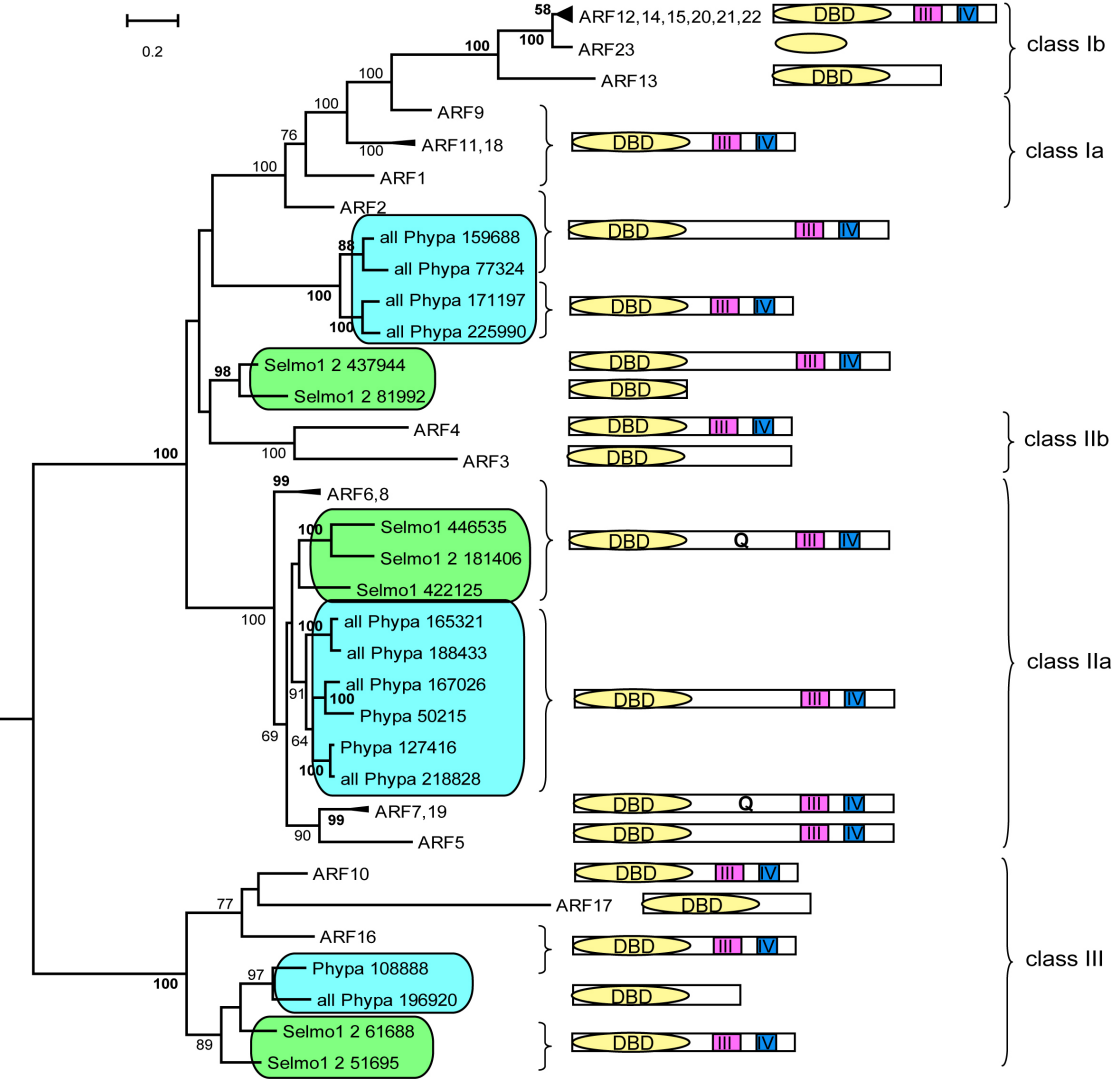
**Phylogenetic relationship of *A. thaliana*, *S. moellendorffii *and *P. patens *ARF proteins, aligned using DBDs (Maximum Likelihood (ML) method)**. Bootstrap values greater than 49 are recorded. These numbers are given in bold if the NJ value is equal to or higher than the ML value. This applies only to those branches common to both topologies.

To examine evolutionary relationships among *P. patens *(PpARF), *S. moellendorffii *(SmARF) and *A. thaliana *(AtARF) ARF proteins, a rooted phylogenetic tree was constructed from the alignment of the predicted protein sequences of the 12 PpARFs, the 7 SmARFs and the 23 AtARFs predicted to contain a DNA binding domain (DBD). All 42 *ARF *genes analysed could be grouped into five major classes (Figure 2). In addition to the five previously described classes of ARFs [35], we detected an additional cluster of four *P. patens *genes and one cluster of two *S. moellendorffii *genes, each with good bootstrap support. Six PpARFs are similar to subclass IIa (AtARF5-8 and 19) and two are similar to class III (AtARF10, 16 and 17). PpARFs, therefore, fell into one of three classes (Figure 2). As in *P. patens*, *S. moellendorffii *has representatives of subclass IIa (three genes) and class III (two genes). The *S. moellendorffii- *and *P. patens-*specific subclasses are not monophyletic.

**Figure 3:**
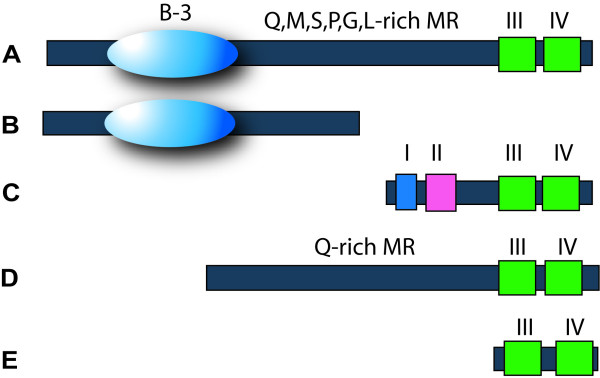
**Domain structures of the Aux/IAA and ARF families found in *A. thaliana*, *S. moellendorffii *and *P. patens***. Canonical, full length ARFs (A); C-terminally truncated ARFs (B); Aux/IAAs (C); N-terminally truncated ARFs (D); *S. moellendorffii*-specific ARF with a large N-terminal truncation (E).

In addition, *P. patens *and *S. moellendorffii *each encode one C-terminally truncated ARF with no CTD (Figure 2, Figure 3, Table 1). Flowering plants encode more CTD-truncated ARFs. This trend is seen in *A. thaliana *(4 out of 23 ARFs), *O. sativa *(6 out of 25 ARFs) and *P. trichocarpa *(6 out of 39 ARFs) (Table 1). Diversification of CTD-truncated ARFs in flowering plants suggests a role for auxin-independent regulation of auxin responsive genes.

#### Full-length ARF transcriptional activators

In *A. thaliana*, the first transcriptional response to exogenously applied auxin is a rapid up-regulation of auxin-responsive genes [4]. The so-called middle regions (MRs) of five AtARFs of sub-class IIa (AtARFs 5, 6, 7, 8 and 19) mediate this transcription [11]. All five of these MRs (as defined by the region between the CTD and DBD) are significantly longer than those of all other ARFs, with the exception of AtARF2 [40]. PpARFs and SmARFs of class IIa also contain an extended MR (Additional file 8 and 9). A second feature of the MRs of those AtARFs which function as transcriptional activators is a relatively high proportion of glutamine residues (except for AtARF5) (Additional file 9 and 10). The MRs of canonical PpARFs of this group contain fewer glutamine residues than their vascular plant counterparts at between 7.8 and 10% of all amino acid residues, compared to between 17.1 and 22.3 for the Q-rich ARFs of *A. thaliana*. PpARFs are unidentifiable as Q-rich both by the normalized amino acid frequency used for Additional file 10 and by a PROSITE domain search. Nevertheless, these MRs all contain a higher proportion of glutamine residues than all but two of the repressor AtARFs (Additional file 9). Given the character states of the MR length (Additional file 8) and glutamine content (Additional file 10) in the phylogenetic tree, a single gain of the domain (basal to the cluster starting with AtARF7 and 19) seems to have occurred. The MR seems to have been secondarily reduced in one SmARF (Selmo1_2_438333) and secondarily expanded in AtARF2. The subsequent enrichment of the MR with glutamine residues apparently evolved several times independently within the genes containing the prolonged MR. *S. moellendorffii *contains three class IIa canonical ARF transcriptional activators. These proteins all contain an extended, Q-rich MR. The simultaneous appearance of an LxLxL motif in *S. moellendorffii *Aux/IAAs allows the possibility that this motif co-evolved with the appearance of canonical Q-rich ARFs.

The exogenous application of auxin to *P. patens *has been shown to have only a weak effect on the expression of transgenic flowering plant auxin-responsive markers [28,29,41]. In contrast, auxin-responsive transcription in *A. thaliana *is observed rapidly, and at relatively low auxin concentrations [4,42]. The slower response in *P. patens *could be due to a number of reasons relating either to an inability of the moss to recognize auxin-responsive flowering plant promoter elements, or to a slower auxin response in *P. patens per se*. Direct experimental evidence is needed if we are to state firmly that there is indeed a slower auxin response in *P. patens*, and that this is due to a relatively weak activation of gene transcription by ARFs. However, the observations that i) the LxLxPP motif of Aux/IAA domain I has been gradually replaced by an LxLxL motif in most flowering plants Aux/IAAs, ii) Q-rich ARFs and the LxLxL EAR-like domain appear together in *S. moellendorffii*, iii) mutations in the canonical LxLxL motif confer weaker transcriptional repression in *A. thaliana *[14], and iv) there is a relatively slow transcriptional response of *P. patens *to auxin together lead us to hypothesize that the Q-enriched subclass IIa ARFs of *P. patens *are moderate rather than strong transcriptional activators.

#### N-terminally truncated ARFs are candidate trans-acting ARF regulators

**Figure 4:**
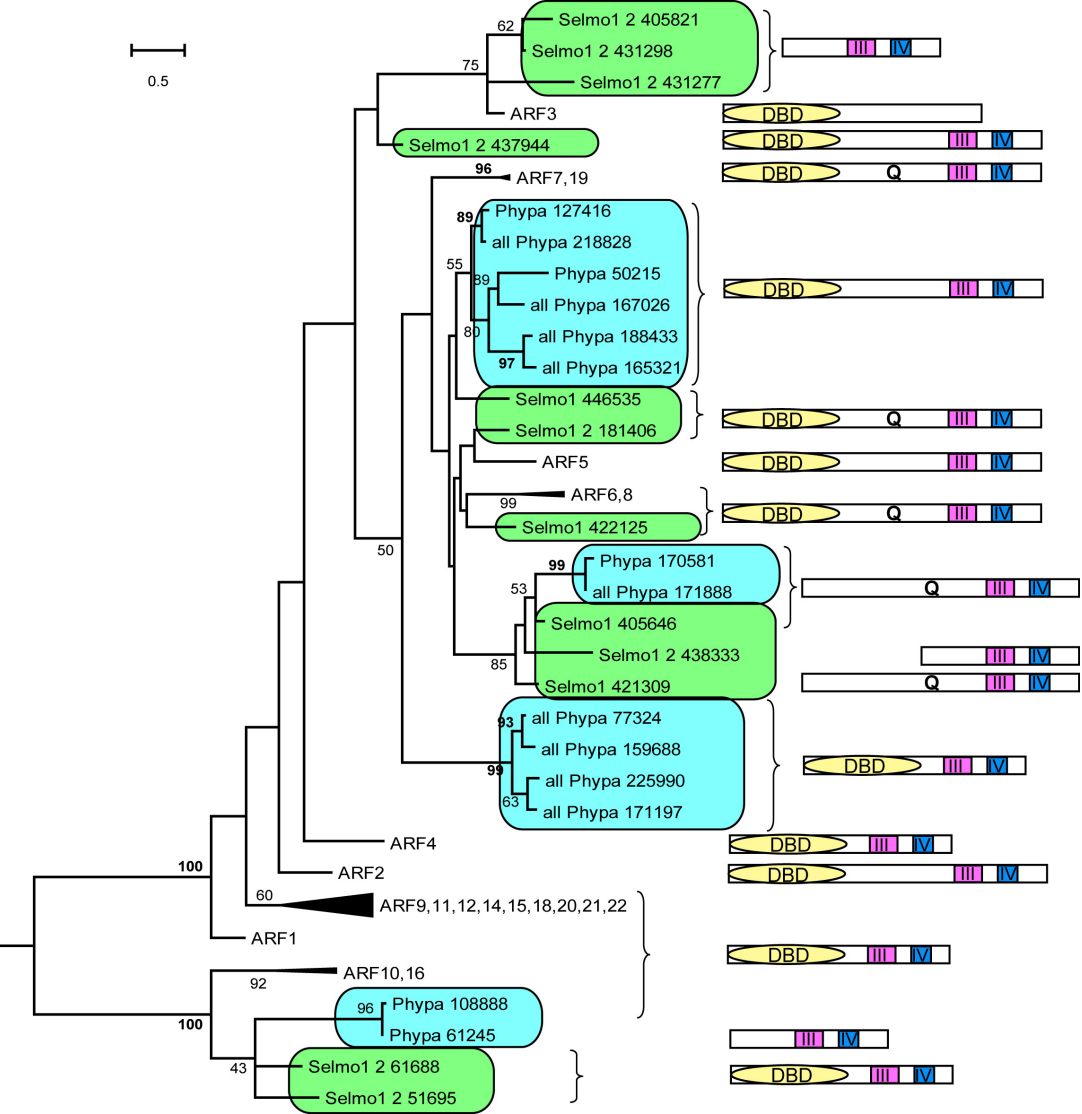
**Phylogenetic relationship of *A. thaliana*, *S. moellendorffii *and *P. patens *ARF proteins, aligned using CTDs (Maximum Likelihood (ML) method)**. Bootstrap values greater than 49 are recorded. These numbers are given in bold if the NJ value is equal to or higher than the ML value. This applies only to those branches common to both topologies.

**Figure 5:**
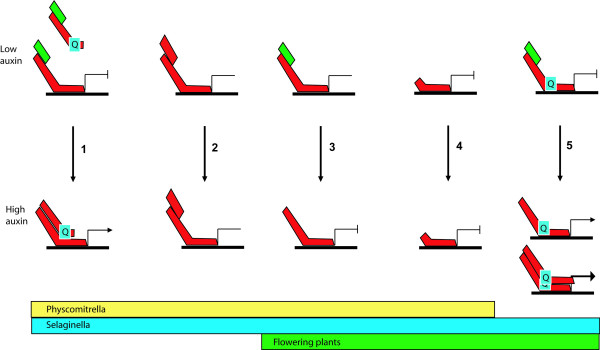
**Model of auxin signalling in *A. thaliana*, *S. moellendorffii *and *P. patens***. (1) In *P. patens *and *S. moellendorffii*, N-terminally truncated Q-rich MRs are predicted to dimerise with full length ARFs to mediate transcription in the presence of auxin. In the absence of auxin, Aux/IAAs inhibit this interaction. Dimirization between other canonical ARFs is also possible. (2) *A. thaliana*, *S. moellendorffii *and *P. patens *full-length ARFs have a MR that is not Q-rich, which can dimerize with either truncated regulatory ARFs in an auxin-independent manner (in *P. patens *and *S. moellendorffii*), (3) or with Aux/IAAs in an auxin dependent manner (*A. thaliana*, *S. moellendorffii *and *P. patens*). (4) *A. thaliana*, *S. moellendorffii *and *P. patens *C-terminally truncated ARFs mediate transcription in an auxin-independent manner. (5) *A. thaliana *and *S. moellendorffii *ARFs with Q-enrichment in their MR. These ARFs directly activate expression of auxin responsive genes. ARF activators also dimerize and potentiate the activation of auxin responsibe genes. These ARFs are regulated by Aux/IAAs in an auxin-dependent manner.

Two proteins encoded by the *P. patens *genome, Phypa_171888 and Phypa_170581, contain both a CTD and an extended Q-rich MR (at 14.0 and 14.5% Q) (Figure 3, 4). However, neither protein contains a recognizable DNA-binding domain (DBD); both gene models were manually checked for accuracy. This phylogenetic analysis placed both truncated *P. patens *proteins in class IIa with AtARF transcriptional activators 6, 7, 19, 5 and 8. The genome of *S. moellendorffii *also encodes two DBD-truncated ARFs in a monophyletic group with N-terminally truncated *P. patens *ARFs. Artificially truncated AtARF5, 6, 7 and 8 proteins (with their DNA-binding domains removed) have previously been shown to activate strongly (15- to 20-fold) transcription of an auxin-responsive reporter gene by dimerizing with canonical ARFs [11]. Therefore, it is possible that the activation of an auxin response in *P. patens *could be relayed by a CTD-dependent heterodimerization between a DBD-truncated ARF and a canonical ARF (Figure 5[1]).

The presence of DBD-truncated Q-rich ARFs allows an alternative transcriptional control, alongside the evolution of a functional motif in domain I of Aux/IAAs. Such an N-terminal truncation enables the spatial separation of transcription-activating MRs and DBDs by the competitive inhibition of ARF CTDs by Aux/IAAs (Figure 5[1]). A functional domain I-motif would not be necessary for such inhibition. Since such an inhibitory mechanism is not able to separate the DNA-binding and activation domains present in a single ARF, we hypothesize that a strong selection pressure on domain I of Aux/IAAs for the efficient recruitment of transcriptional co-repressors could have been a feature of Aux/IAA evolution after the appearance of canonical Q-rich ARFs. This hypothesis would predict that at least two mechanisms have evolved through which the evolution of a strong ARF activation domain has been accommodated: firstly, the appearance of a strong Aux/IAA repressor domain, as seen in flowering plants, and secondly, the spatial separation of the ARF activation domain from the DNA-binding domain, as seen in *P. patens*. Notably, *S. moellendorffii *is predicted to employ both.

A second group of proteins with an N-terminal truncation is encoded by the genomes of *P. patens *and *S. moellendorffii *(Table 1, Figure 3, Figure 5[2]). Here the truncation is larger, and the encoded proteins are predicted to have neither a DBD-domain, nor a middle region. We propose that proteins of this group act as auxin-independent competitive inhibitors of ARF dimerization, inhibiting both potentiation (via ARF-ARF dimerization) and repression (via ARF-Aux/IAA dimerization) of the auxin response.

### Evolution of ARF activators

To test whether positive selection, and therefore possible neofunctionalization, has driven evolution within the extended ARF MR of class IIa, we compared the relative rates of synonymous and non-synonomous substitutions in full-length coding sequences from all ARFs of two fully-sequenced dicotyledonous species: *A. thaliana *and *P. trichocarpa*. A likelihood ratio test (LRT) was applied to selected ARF sequences from *A. thaliana *and *P. trichocarpa *(Additional file 6, B). The maximum likelihood estimates (MLEs) of parameters under model M2a and M8 are listed in Table 3, together with the sites inferred to be under positive selection by the Bayes empirical Bayes (BEB) approach. For node ARF12, both M2a and M8 have significantly higher likelihood values than their corresponding null models M1a and M7, suggesting the presence of sites under positive selection. For node ARF7, M8 (but not M2a) had significantly higher likelihood values than its corresponding null model M7 (Table 3). Consequently, this model identified 24 sites under positive selection (21 sites are presented in Additional file 11), all within the extended MR (Table 3), supporting the hypothesis that positive selection within the MR plays a role in neofunctionalization, possibly directly influencing the acquisition of a transcriptional activation function. It is however possible that positive selection influences an unrelated function of the MR. For example, the MR may influence protein stability, as is the case for the MR of ARF1 [43]. Yet, if the enrichment of glutamine is important for transcriptional activation, the fact that 14 of the 24 sites under positive selection in the MR encode a glutamine in at least one protein clearly argues for the involvement of positive selection in the acquisition of that particular function.

**Table 3:** Sites under PDS in the *A. thaliana*/*P. trichocarpa *ARF gene family: „Site-specific analysis".

Site-specific analyses	n	dN/dS (ω) under M0	2Δℓ M2 vs. M1 (df 2)	2Δℓ M8 vs. M7 (df 2)	Parameter estimates under M8	Positively selected sites under M2 (BEB)	Positively selected sites under M8 (BEB)
**ARF**							
Node ARF2	5	0.183	0.00	0.56	ρ_0 _= 0.957 (ρ_1 _= 0.043) (ρ = 0.431) q = 1.635 ω = 1.09	None	478 540 563 631 758
Node ARF6	7	0.122	0.00	1.04	ρ_0 _= 0.997 (ρ_1 _= 0.003) (ρ = 0.355) q = 1.994 ω = 4.49	None	372464476 479 482 485 539
Node ARF7	6	0.154	0.00	13.78**	ρ_0 _= 0.973 (ρ_1 _= 0.027) (ρ = 0.409) q = 1.814 ω = 3.086	None	460 526 532534 539 542*544 *546 550 556 557 558 559 561 562 566568 569 570 574599 608 677 785
Node ARF10	8	0.137	0.00	0.64	ρ_0 _= 0.998 (ρ_1 _= 0.016) (ρ = 0.413) q = 1.988 ω = 2.69	None	96 449 486*570*
Node ARF11	5	0.151	0.00	1.74	ρ_0 _= 0.932 (ρ_1 _= 0.068) (ρ = 0.528) q = 2.885 ω = 1.00	None	6 334 370 372 376 383 399 412 413 478 529
Node ARF12	6	0.566	19.42**	19.4**	ρ_0 _= 0.982 (ρ_1 _= 0.018) (ρ = 0.056) q = 0.054 ω = 10.50	175335389 433 444447 527 **567 569 570 ***572*	175*335 *351 359 389 433 *444 447 *527 **567 569 570 572**

Each comparison has n sequences, dN/dS is average ratio over sites under a codon model with one ω. Bold underline, PP ≥ 0.99 of being under positive selection; bold, 0.99>PP ≥ 0.95; italic, 0.95>PP ≥ 0.90; underline, 0.90>PP ≥ 0.70; normal, 0.70>PP > 0.50

ARF7 and ARF19 dimerize with Aux/IAAs to regulate the expression of partially overlapping sets of auxin-responsive genes in the control of lateral root development and gravitropism [44]. However, ARFs do not only dimerize with Aux/IAAs. In Arabidopsis, a member of a second class of transcription factors, MYB77, interacts with the CTD ARF7 to control auxin-responsive gene expression and lateral root number [45]. Therefore a third interaction, besides DNA or Aux/IAA interaction, influences ARF evolution. As the interaction between MYB77 and ARFs occurs with the ARF CTD, it cannot explain positive selection within the proteins' MR. It does, however, represent a precedent for Aux/IAA independent protein-protein interactions (and possible subsequent post-translational modification) influencing protein function within the ARF7 node, and the presence of, as yet unconsidered, evolutionary pressures influencing ARF function.

### Evolution of ARFs which lack a Q-rich middle region

In contrast to the relatively constant numbers of class IIa ARF transcriptional activators encoded by the genomes of *P. patens, S. moellendorffii *and *A. thaliana *(six, three and five respectively), the number of ARF repressors has increased from five and four in *P. patens *and *S. moellendorffii *to fourteen in *A. thaliana*. There is only one *P. patens*-specific and one *S. moellendorffii*-specific ARF class, whilst there are three flowering plant-specific sub-classes (class Ia, class Ib and class IIb) indicating that evolution within flowering plants has favoured strongly the diversification of auxin-regulated repressor ARFs (Figure 2).

Two polyphyletic groups of ARF lacking a Q-rich region can be differentiated: those with a CTD and those without. This suggests at least two distinct mechanisms of transcriptional regulation. ARFs which lack a CTD are responsible for the auxin-independent (or basal) regulation of auxin-responsive genes (Figure 5[4]). These ARFs cannot interact with Aux/IAAs and therefore their transcriptional activity is independent of cellular auxin concentration. However, identity within their DNA-binding domain suggests they are able to bind to auxin responsive promoter elements. The second type of ARF has a CTD and is, at least according to the accepted paradigm, able to dimerize with Aux/IAAs [9,18,46] (Figure 3, 5[3]). Phosphorylation of ARF2 (a full length ARF) by BIN2, a kinase involved in brassinosteroid-dependent transcription decreases its ability to bind DNA [47]. This path for crosstalk between two hormone signalling pathways (auxin and brassinosteroid) represents a precedent for ARF repressors to perform in other signalling functions.

After analysis of all genes encoding *A. thaliana *ARF transcriptional repressors, positive selection was only observed in the ARF12 node (Class Ib), where little is known about protein function (Additional file 6, B). In this class, single knockouts do not show obvious aberrant phenotypes [44], and the generation of double knockout lines has been hampered by the genes' close proximity on chromosome 1.

Class Ib ARFs are absent from the *P. trichocarpa *and *O. sativa *genome, raising the possibility of a specific role within the order Brassicales [27,35]. Positive selection does not prove the acquisition of novel and specific function in the class Ib ARFs of *A. thaliana*. However, together with the subgroup's rapid and significant diversification, followed by the retention of duplicated genes, it suggests neofunctionalization. Any putative new function is also likely to be related to the amino acid residues under positive selection in the middle region of the protein, possibly facilitating new protein-protein interactions, protein stability, or post-translational modifications.

### Auxin-independent regulation of ARF activity

ARFs without a CTD cannot dimerize with Aux/IAAs and are therefore not expected to be regulated directly by auxin. But is auxin-independent ARF signalling relevant to flowering plants, or is ARF function dependent on a functional CTD? CTD-deficient *arf *mutants do indeed have aberrant phenotypes. Four ARF proteins which lack a CTD are predicted to be encoded by the *A. thaliana *genome. Of these, *ARF3 *mutants show pleiotropic effects in flower development [48]. Plants expressing a miRNA-resistant version of a second CTD-deficient ARF, *ARF17*, have increased *ARF17 *mRNA levels and display dramatic developmental defects. These include embryo and emerging leaf symmetry anomalies, leaf shape defects, premature inflorescence development, altered phyllotaxy, reduced petal size, abnormal stamens, sterility, and root growth defects [49]. The search for alternative mechanisms of ARF regulation has centred on small RNAs. Two *P. patens ARF *transcripts (*Phypa_159688 *and *Phypa_171197*), both encoding full-length ARFs (Figure 4), have been identified as targets of small RNAs [50]. Regulation of ARFs by miRNA in *A. thaliana *can be considered as auxin-independent because auxin treatment does not alter appreciably *miR160*, *miR164*, and *miR167 *accumulation, at least in seedlings [49]. In *A. thaliana*, mRNAs encoding two out of the four ARFs which have no CTD have also been identified as targets of small RNAs: *ARF3 *is the target of *AtTAS3a-c *and *ARF17 *is the target of *miR160 *[50]. Small RNAs do not only target transcripts of ARFs without a CTD, but also Aux/IAA-binding ARFs. The regulation of ARF activity is therefore complex and involves the integration of auxin-dependent and auxin-independent mechanisms (Figure 5). miRNAs are also potentially important regulators of cross-talk between auxin and other signalling pathways, for example between auxin and abscisic acid [51].

Auxin-independent, cell-dependent regulation of auxin signalling activity has previously been identified as an important factor in plant development [52]. Indeed, endogenous small regulatory RNAs seem to play a relatively important role in the regulation of *ARF *gene expression [53]. For example, in *A. thaliana *the expression pattern of both *ARF6 *and *ARF8 *(involved in female and male reproductive organ development) is controlled by *miR167*, with *miRNA160 *also involved in the control of *ARF *expression in *P. patens *and *A. thaliana *as well as in *S. moellendorffii*, suggesting a conserved mechanism of *ARF *post-transcriptional regulation [52,54]. The *P. patens *genome encodes a surprisingly diverse population of miRNAs. However, in contrast to *ARF *and *Aux/IAA *genes, the number of miRNAs conserved between *P. patens *and *A. thaliana *is relatively large [55].

### Primary auxin response genes

Primary auxin response genes (those genes whose expression is directly regulated by ARFs) can be grouped into three major families: *Aux/IAAs*, *GH3s *and *SAURs*. Recently, transcription of certain *LOB domain *(*LBD*) genes has also been shown to be rapidly and specifically up-regulated by auxin [4,56]. All four of these major gene families are represented in the genomes of *P. patens *and *S. moellendorffii*. However, a detailed analysis of their response to auxin application is precluded by the lack of global transcriptional data from these species.

Microarray analysis has showed that, in *A. thaliana*, only the transcription of group II *GH3 *genes (which encode auxin conjugating enzymes) is regulated by auxin [4] Similarly, in *O. sativa*, the transcription of GH3 genes which were most strongly up-regulated in response to auxin treatment also belong to group II [57]. The *P. patens *genome contains two genes that are homologous to the *GH3 *family of flowering plants. Both conjugate IAA to amino acids, with PpGH3-2 showing a far broader range of substrate specificity than PpGH3-1 [58]. Surprisingly, the moss *GH3 *genes form a common clade with the group I genes of *A. thaliana*, and not with those encoding the auxin conjugating enzymes of group II. Furthermore, the clades are separated by a relatively high genetic distance, suggesting that they diverged a relatively long time ago (Additional file 12). Auxin application increases transcription of specific flowering plant *GH3 *genes of group II. This increase has never been demonstrated in *P. patens *[59]. The genome of *S. moellendorffii *is predicted to encode one group II GH3 enzyme, and one protein belonging to group I (Additional file 12). The remaining 19 *SmGH3 *genes cannot be clearly assigned based on phylogeny. The transcriptional response to auxin of these genes has never been tested.

*P. patens *GH3 enzymes are nevertheless able to conjugate auxin. Direct measurements of auxin conjugates in moss plants have give valuable insights into the developmental role of auxin conjugation by GH3s. *P. patens *plants lacking both GH3 enzymes, when grown on IAA, still conjugate auxin. These results suggest other classes of enzymes may also conjugate auxin in *P. patens *[58].

Based on phylogeny *P. patens *GH3s are more closely related to GH3-11 of *A. thaliana*, which catalyses the synthesis of jasmonic acid conjugates [58,60]. *P. patens *plants lacking the GH3-2 gene show an increased sensitivity to high jasmonic acid concentrations, suggesting a potential role for jasmonic acid conjugation as well for this enzyme [58]. A broad substrate specificity of GH3-2 in *P. patens *could suggest that the enzyme has retained this characteristic from the common ancestor of all land plants.

In flowering plants, *SAUR *genes are a diverse family of unknown function with differing responsiveness to auxin [4]. The *P. patens *genome contains 18 SAUR genes (*A. thaliana *approximately 70), which cluster in two groups with low bootstrap support (Additional file 13). All *AtSAUR *genes of group A are auxin-responsive [4,62]. This group shows relatively high similarity to nine *PpSAUR *genes (albeit with low bootstrap support) (Additional file 13) and therefore could participate in the auxin response in *P. patens*. The LOB domain family of transcription factors also contains important auxin-responsive signalling proteins. In *P. patens*, the LBD gene family has 17 members, forming five clades (Additional file 14). One clade, encoding four LBDs (Phypa_18666, 7278, 25219 and 48669), is monophyletic with important auxin-responsive regulators of lateral root formation in *A. thaliana*, LBD16 and 29 [56], and therefore represents candidates for *P. patens *auxin primary response, an attractive target for future research.

## Conclusion

It is clear that auxin signalling is responsible for many aspects of vascular plant growth and development. In this manuscript, we demonstrate that the genome of *P. patens *encodes all of the basic components necessary for an auxin response. We also suggest that the evolution of an alternative, competitive mechanism of transcriptional control in *P. patens*, involving the truncation of ARF transcriptional activators, substitutes for a mechanism which, in *S. moellendorffii *and flowering plants, confers a rapid auxin response.

However, without a systematic analysis of the auxin transcriptional response in *P. patens *and *S. moellendorffii *it remains difficult to assess (i) whether these plants are capable of rapidly synthesizing specific mRNAs in response to auxin in the same manner as flowering plants, and (ii) the role any such response plays in auxin homeostasis and plant development.

It is, however, clear that an expansion of the *Aux/IAA *gene family accounts for much of the diversification of auxin signalling proteins in flowering plants. Furthermore, the smaller size of many gene families relevant to auxin signalling in *P. patens *is probably correlated to the lower structural complexity of this plant. This correlation is especially pronounced in *Aux/IAA *gene families.

Auxin and its polar transport are crucial factors in flowering plant development, and have come to direct many processes which are not relevant to mosses such as apical dominance, formation and maintenance of shoot and root apical meristems and vascular differentiation. Mosses nevertheless require auxin for cell differentiation and division. Understanding the differences in the underlying mechanisms of auxin signalling, which drive these different physiological processes, and of their evolutionary relationship, will be a fascinating challenge for the future.

## Methods

### Candidate gene family member selection and curation

To define and extract the *ARF*, *Aux/IAA *and *TIR1 *gene families we screened the published genomes of *A. thaliana *(TAIR7; ), *O. sativa *(Osa1 version 5.0; ), *P. trichocarpa *(Poptr1_1;  ), *S. bicolor *(Sbi1.4; ), *V. vinifera *(Vitis_vinifera_v1; ), *G. max *(Glyma0.1b.pep.fa.gz; ) and *P. patens *(Phypa1_1; ) by BLASTP against a database containing all (predicted) proteins of the respective organisms. As queries, the known members of the *A. thaliana *gene families were used. For *ARF *queries, At1g35240, At1g77850 and At5g60450 were selected; for *Aux/IAA*, At1g04550, At2g01200 and At4g14560; and for *TIR1*, At3g62980 and At5g49980. Based on the protein domain architecture of the *A. thaliana *proteins, BLAST results were inspected manually to determine query specific filtering criteria. For *ARF *sequences, we required that 30% of amino acids be identical and 50% of aligned amino acid sites be shared; for *Aux/IAA *sequences, we used an E-value threshold of 1E-40, and 1E-62 for *TIR1*. The *S. moellendorfii *candidate gene family members were detected using BLASTP against the filtered models 2 predicted proteins using the filtering criteria mentioned above. All candidate loci were manually inspected using the JGI genome browser  and curated to select the "optimal" gene model. Additionally, the genomic contexts (~40 kbp) of highly conserved gene model pairs were compared to exclude redundancies due to gene models representing loci from the two sequenced haplotypes. Furthermore, the *P. patens *genome v1.1 was screened for additional, as yet undetected, gene family members using Exonerate [63]. All detected *P. patens *candidate loci were inspected manually using the cosmoss.org genome browser . Under consideration of all available cDNA, EST and protein evidences the "optimal" predicted gene model was derived for each locus. To reduce complexity and maintain readability of the resulting phylogenetic trees, further analysis only included the candidate proteins from *P. patens*, *S. moellendorfii *and *A. thaliana*. ARF and Aux/IAA *P. patens *protein IDs: 108888, 127416, 170581, 50215, 61245, 164608, 219923, 159688, 165321, 167026, 171197, 171888, 188433, 196920, 218828, 225990, 77324 and BAB71765. ARF and Aux/IAA *S. moellendorfii *protein IDs: 431277, 431298, 405821, 438333, 181406, 61688, 51695, 437944, 81992, 406764, 412634, 26861, 405646, 421309, 446535, and 422125.

### Domain annotation and multiple sequence alignments

Protein domain architectures of the *ARF *and *Aux/IAA *candidate hits were annotated using the Pfam [64] Hidden Markov Profiles (HMMs) PF02362.12 (B3, representing the DBD), PF06507.4 (Auxin_resp), PF02309.7 (AUX_IAA, representing the CTD) and the PROSITE [65] profile PS50962 (IAA_ARF) using the hmmpfam and the ps_scan tools and applying each domain profile's "trusted cutoff" as filtering criteria. To extract CTD domain region from both, *ARFs *and *Aux/IAAs *(CTD+), the FASTA output option of ps_scan was used. CTD+ domain sequences were aligned with MAFFT L-INSI [66], ProbCons, Muscle and T-coffee and subsequently combined into an optimal alignment using the combiner function of T-coffee [67]. Full-length multiple sequence alignments (MSAs) were calculated using Dialign [68]. Full-length MSAs including the protein domain annotation were visualized and manually inspected and curated using the Jalview [69] alignment editor. In order to generate data for the domain-based phylogenies, the full-length MSAs were clipped to either the N-terminal DNA-binding (DBD; extending the B3 + Aux_resp domain matches) or the C-terminal interaction domain (CTD; extending the Aux_IAA domain matches) regions, according to the domain annotation and alignment quality. Proteins missing both individual domains were discarded and the clipped MSAs were realigned using the MAFFT [66] L-INSI algorithm.

### Phylogenetic analyses

Bayesian inference was performed using MrBayes for the clipped *Aux/IAA *and the CTD+ MSA with 2 runs with a mixed model prior, a proportion of invariable sites and gamma distribution for a maximum of 2,000,000 using a temperature of 0.2 and a sampling rate of 5. Maximum Likelihood (ML) and Neighbor Joining (NJ) phylogenies were calculated for the full-length *Aux/IAA *MSA and the clipped MSAs for the DBD, the *ARF*-specific CTD and the CTD including the *Aux/IAA*s (CTD+). Bootstrapped (100×) NJ trees were calculated using a modified version of the quicktree software [70], with the Scoredist [71] matrix. ProtTest [72] was used to select the most appropriate evolutionary model for ML inference (DBD:JTT+G; CTD:JTT+G; *Aux/IAA*:JTT+G+F). Bootstrapped (100×) best-known likelihood topologies were calculated using the parallelized version of RAxML [73]. Generally, phylogenetic trees were rooted by midpoint-rooting. The CTD, as the common feature of both families, was used to root the ARF and AUX/IAA trees. To infer the history of duplications and losses, the CTD+ phylogeny was reconciled with Notung [74], as used in [75-77] applying the species tree (Phypa, (Selmo, Arath)).

### Character state analyses

The MR was defined as the region between DBD and CTD, or in case of a lack of the DBD as the region from the N-terminus to the start of the CTD. The length of the MR was transformed into a continuous character matrix comprising eight characters. Q-rich regions were represented by the amino acid frequency normalized to the length of the MR. The resulting character matrix was analyzed using the Mesquite [78] analysis tool "Trace Character History" on the basis of the Notung reconciled CTD+ MrBayes phylogeny. Nucleotide alignments of coding sequences were performed on the basis of protein alignments. The protein sequences were aligned with MAFFT [69]. DAMBE 4.5.55 [79] was used to translate protein alignments to nucleotide alignments.

### Statistical tests for positive selection

We applied the codon-based substitution model of Yang et al. [80] to identify amino acid sites under positive selection using PAML3.14 [81]. First, we ran a test for the existence of sites with a dN/dS ratio > 1 by using a likelihood ratio test (LRT) to compare null models M1a and M7(beta) (that do not allow for sites with dN/dS >1) with alternative models M2a (PositiveSelection) and M8(beta&ω). If the LRT difference was statistically significant we identified the sites that were under positive selection. Naïve empirical Bayes (NEB) and Bayes empirical Bayes (BEB) approaches were used [82] to calculate the posterior probability that each site belongs to a particular site class. Sites with high posterior probabilities from the class with ω>1 were inferred to be under positive selection.

### Microarray experiments

The microarray gene expression data for paralogous pairs of *Aux/IAA *genes were analyzed in 63 diverse samples [32] (in our analysis, we included only data generated from wild type plants). gcRMA normalized data were used [32]. Three biological replications were used to generate the data sets. To identify which components contribute to expression pattern divergence within each duplicate pair, the two-way ANOVA used by Duarte et al. [31] to partition the gene (G), sample (S), and gene by sample interaction (GxS) effects was extended to all 63 microarray samples. Analysis was done using Statistica 5.0.

## Abbreviations

ARF: AUXIN RESPONSIVE FACTOR; AuxREs: Auxin responsive elements; Aux/IAA: AUXIN/INDOLE-3-ACETIC ACID; BEB: Bayes empirical Bayes; CTD: C-terminal dimerization; DBD: NDA binding domain; EAR: ERF-associated amphiphilic repression; ERF: ethylene response factor; HMMs: Hidden Marsov Profiles; LBD: LOB domain; LRT: likelihood ratio test; MLEs: maximum likelihood estimates; MSAs: multiple sequence alignments; MR: middle region; Q: glutamine; NEB: Naïve empirical Bayes; NJ: Neighbor Joining; TPL: TOPLESS.

## Authors' contributions

IAP, WT, DL, RR, SAR, KP designed research. IAP and DL performed research. IAP, DL, SAR and MP analyzed data. WT and IAP wrote the paper.

## Supplementary Material

Additional file 1 **Amino acid sequence alignment of Aux/IAA proteins of *A. thaliana*, *S. moellendorffii *and *P. patens *across conserved domain I**. An LxLxL motif is encoded by *Aux/IAA*s of *A. thaliana *and *S. moellendorffii*, but not of *P. patens*. Click here for file

Additional file 2 **Phylogenetic relationship of *A. thaliana *and *P. patens *TOPLESS-like transcriptional co-repressors (Neighbor Joining (NJ) method)**. The *P. patens *genome encodes two TOPLESS-like transcriptional co-repressors. Bootstrap values greater than 49 are recorded. Click here for file

Additional file 3 **Amino acid sequence alignment of Aux/IAA proteins of *A. thaliana*, *S. moellendorffii *and *P. patens *domain II**. The core motif of domain II of Aux/IAA proteins was present in all plant species tested. Bootstrap values greater than 49 are recorded. Click here for file

Additional file 4 **Phylogenetic relationship of *A. thaliana *and *P. patens *TIR1-like F-box proteins (Neighbor Joining (NJ) method)**. Four paralogs of the TIR1-family of F-box proteins are present in *P. patens*. Bootstrap values greater than 49 are presented. Click here for file

Additional file 5 **Phylogenetic relationship of *A. thaliana*, *S. moellendorffii *and *P. patens *ARF and Aux/IAA proteins (Bayesian inference)**. To infer the history of duplication and losses among the species tested, the CTD+ phylogeny was reconciled with Notung using the species tree (Phypa, (Selmo, Arath)). Click here for file

Additional file 6 **Phylogeny of *A. thaliana *and *P. trichocarpa *Aux/IAA (A) and ARF (B) proteins**. Boxes identify nodes tested for positive selection. Click here for file

Additional file 7 **Expression pattern of paralogous pairs of *A. thaliana *Aux/IAA genes (A-J)**. gcRMA normalized data were used. Three biological replications were used to generate the data set. The two-way ANOVA was used to partition the gene (G), sample (S) and GxS interaction effects. Click here for file

Additional file 8 **Phylogenetic relationship of *A. thaliana*, *S. moellendorffii *and *P. patens *ARF proteins**. Reconciled tree based on Bayesian inference. Length of middle region was normalized and transformed into a continuous character matrix. Click here for file

Additional file 9 **Detailed comparison of *A. thaliana*, *P. patens *and *S. moellendorffii *ARFs**. Here we present details of the middle region of ARFs, the presence of domain III and IV, amino acid frequency for Q, S, G, P, L, M, the total length of proteins, and the presence of amino acid-rich domains using ScanProsite. Click here for file

Additional file 10 **Phylogenetic relationship of *A. thaliana*, *S. moellendorffii *and *P. patens *ARF proteins**. Reconciled tree based on Bayesian inference. Q-rich regions are represented by the amino acid frequency normalized with the length of the MR. Click here for file

Additional file 11 **ARF protein sequence alignment of the middle regions in the ARF7 node of *A. thaliana *and *P. trichocarpa***. Arrows indicate sites at which positive selection was detected. Boxed amino acids indicate putative phosphorylation motifs. Click here for file

Additional file 12 **Phylogenetic relationship (neighbor-joining (NJ) method) of *A. thaliana*, *S. moellendorffii *and *P. patens *GH3 proteins**. PpGH3s are indicated in light blue. SmGH3s are indicated in light green. Click here for file

Additional file 13 **Phylogenetic relationship (neighbor-joining (NJ) method) of *A. thaliana *and *P. patens *SAUR proteins**. The *P. patens *SAURs are indicated in light blue.*A. thaliana *SAURs transcriptionally up-regulated by auxin are indicated in purple. Click here for file

Additional file 14 **Phylogenetic relationship (neighbor-joining (NJ) method) of *A. thaliana *and *P. patens *LBD proteins**. LBD proteins of *P. patens *are indicated in light green. *A. thaliana *LBDs transcriptionally up-regulated by auxin are indicated in purple. Click here for file
