# Supplementary material for: The evolution of nuclear auxin signalling
Source: BMC Evol Biol. 2009 Jun 3;9:126. doi: 10.1186/1471-2148-9-126 (PMC2708152; doi:10.1186/1471-2148-9-126)
Supplement: Additional file 1 — Amino acid sequence alignment of Aux/IAA proteins of A. thaliana, S. moellendorffii and P. patens across conserved domain I. An LxLxL motif is encoded by Aux/IAAs of A. thaliana and S. moellendorffii, but not of P. patens. [file 1471-2148-9-126-S1.pdf]

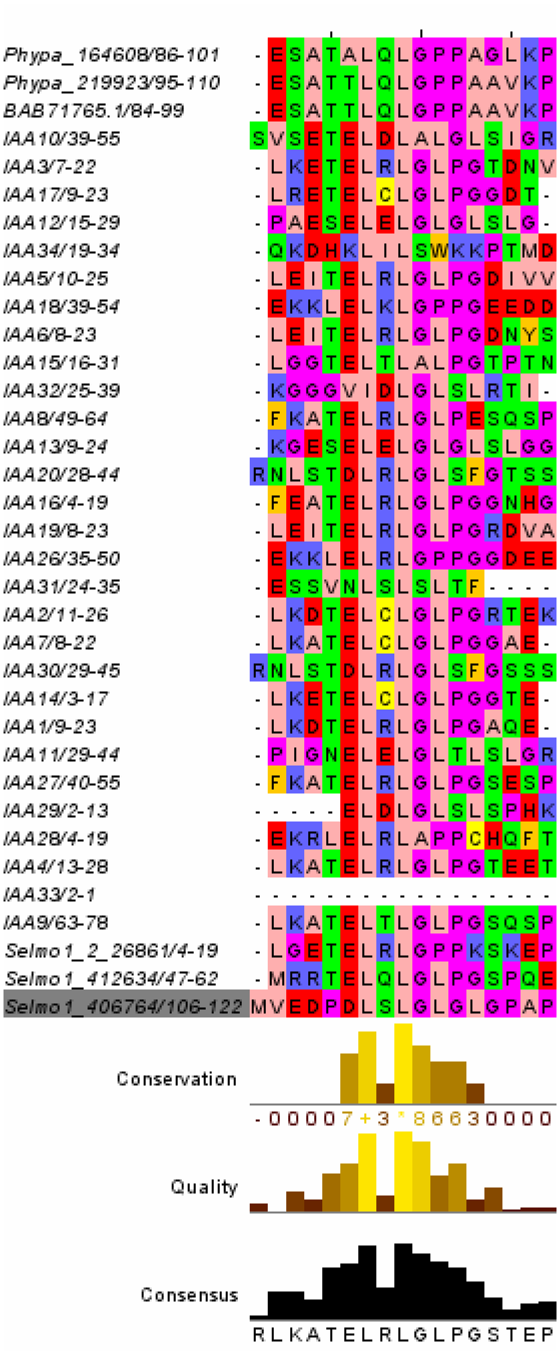

File 1. Amino acid sequence alignment of Aux/IAA proteins of *A. thaliana*, *S. moellendorffii* and *P. patens* across conserved domain I.
