# Supplementary material for: The evolution of nuclear auxin signalling
Source: BMC Evol Biol. 2009 Jun 3;9:126. doi: 10.1186/1471-2148-9-126 (PMC2708152; doi:10.1186/1471-2148-9-126)
Supplement: Additional file 2 — Phylogenetic relationship of A. thaliana and P. patens TOPLESS-like transcriptional co-repressors (Neighbor Joining (NJ) method). The P. patens genome encodes two TOPLESS-like transcriptional co-repressors. Bootstrap values greater than 49 are recorded. [file 1471-2148-9-126-S2.pdf]

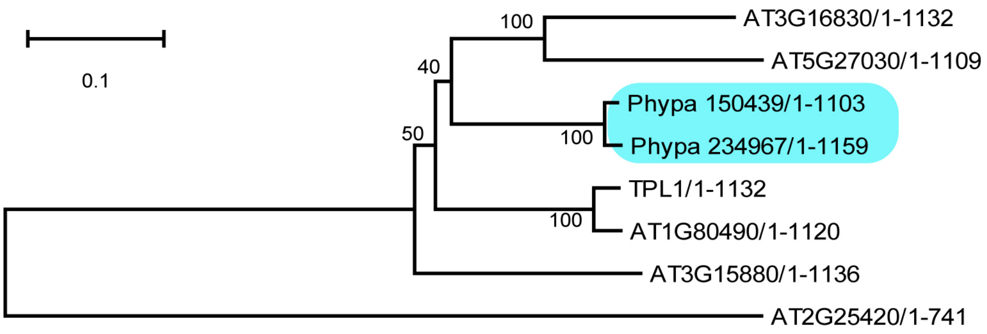

File 2 Phylogenetic relationship of *A. thaliana* and *P. patens* TOPLESS-like transcriptional co-repressors (Neighbor Joining (NJ) method).
