# Supplementary material for: The evolution of nuclear auxin signalling
Source: BMC Evol Biol. 2009 Jun 3;9:126. doi: 10.1186/1471-2148-9-126 (PMC2708152; doi:10.1186/1471-2148-9-126)
Supplement: Additional file 3 — Amino acid sequence alignment of Aux/IAA proteins of A. thaliana, S. moellendorffii and P. patens domain II. The core motif of domain II of Aux/IAA proteins was present in all plant species tested. Bootstrap values greater than 49 are recorded. [file 1471-2148-9-126-S3.pdf]

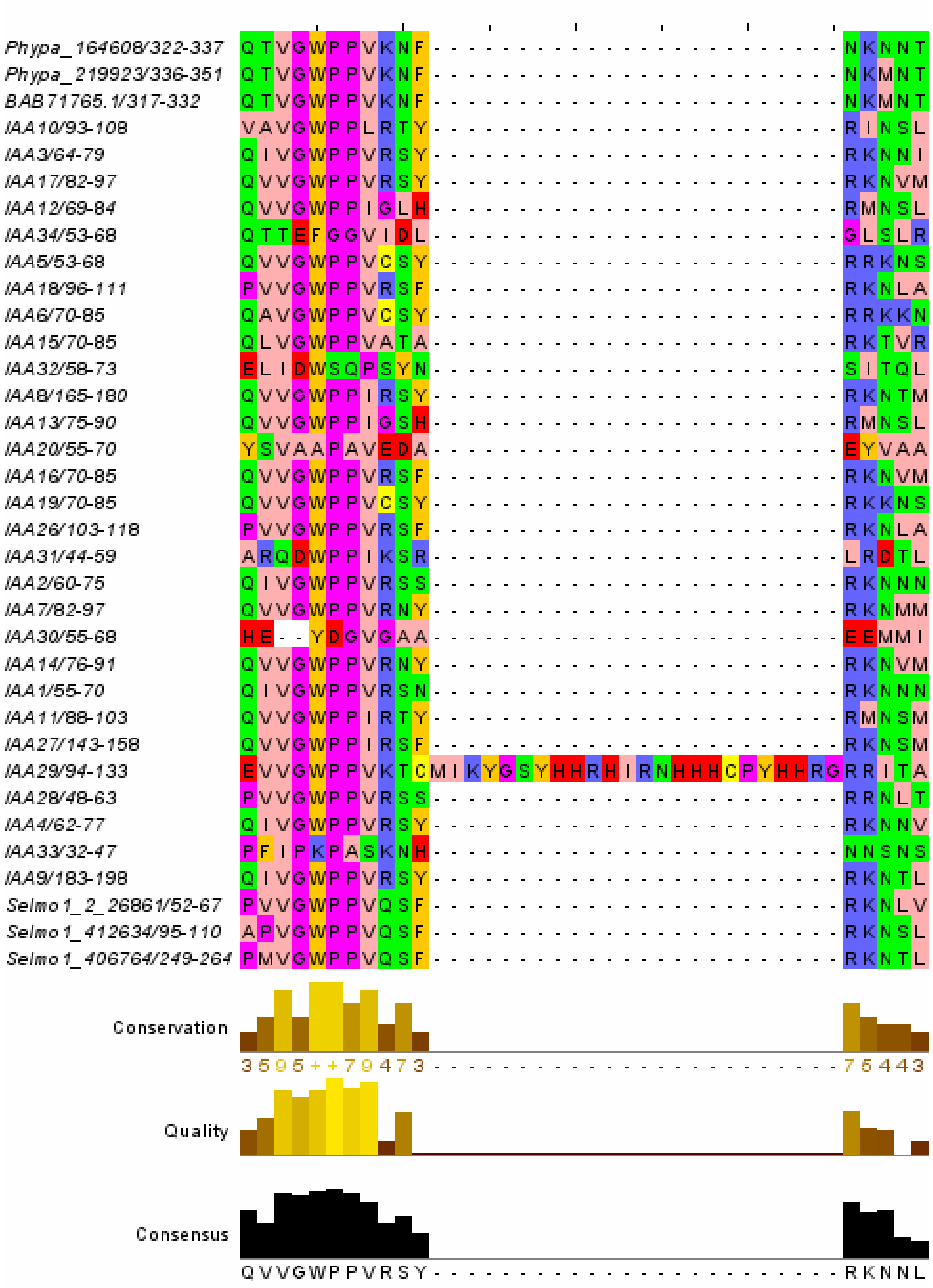

File 3. Amino acid sequence alignment of Aux/IAA proteins of *A. thaliana*, *S. moellendorffii* and *P. patens* domain II.
