# Supplementary material for: The evolution of nuclear auxin signalling
Source: BMC Evol Biol. 2009 Jun 3;9:126. doi: 10.1186/1471-2148-9-126 (PMC2708152; doi:10.1186/1471-2148-9-126)
Supplement: Additional file 4 — Phylogenetic relationship of A. thaliana and P. patens TIR1-like F-box proteins (Neighbor Joining (NJ) method). Four paralogs of the TIR1-family of F-box proteins are present in P. patens. Bootstrap values greater than 49 are presented. [file 1471-2148-9-126-S4.pdf]

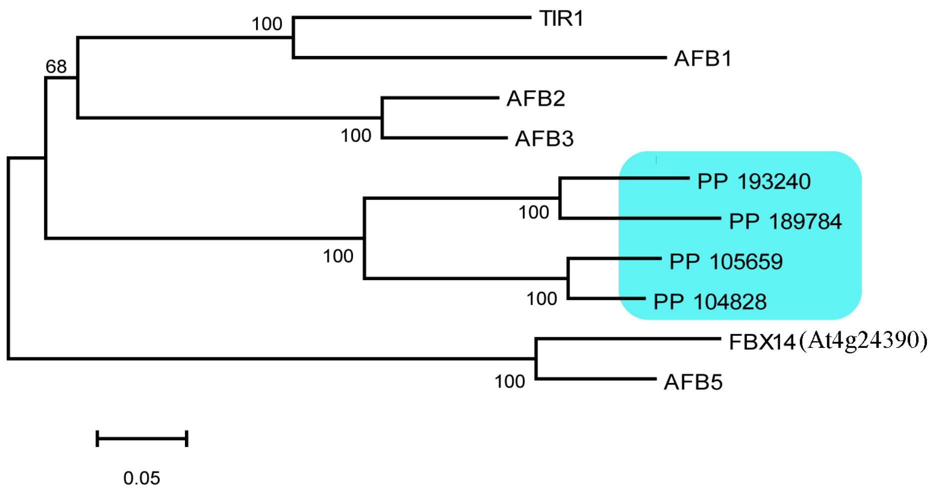

File 4. Phylogenetic relationship of *A. thaliana* and *P. patens* TIR1-like F-box proteins (Neighbor Joining (NJ) method).
