# Supplementary material for: The evolution of nuclear auxin signalling
Source: BMC Evol Biol. 2009 Jun 3;9:126. doi: 10.1186/1471-2148-9-126 (PMC2708152; doi:10.1186/1471-2148-9-126)
Supplement: Additional file 5 — Phylogenetic relationship of A. thaliana, S. moellendorffii and P. patens ARF and Aux/IAA proteins (Bayesian inference). To infer the history of duplication and losses among the species tested, the CTD+ phylogeny was reconciled with Notung using the species tree (Phypa, (Selmo, Arath)). [file 1471-2148-9-126-S5.pdf]

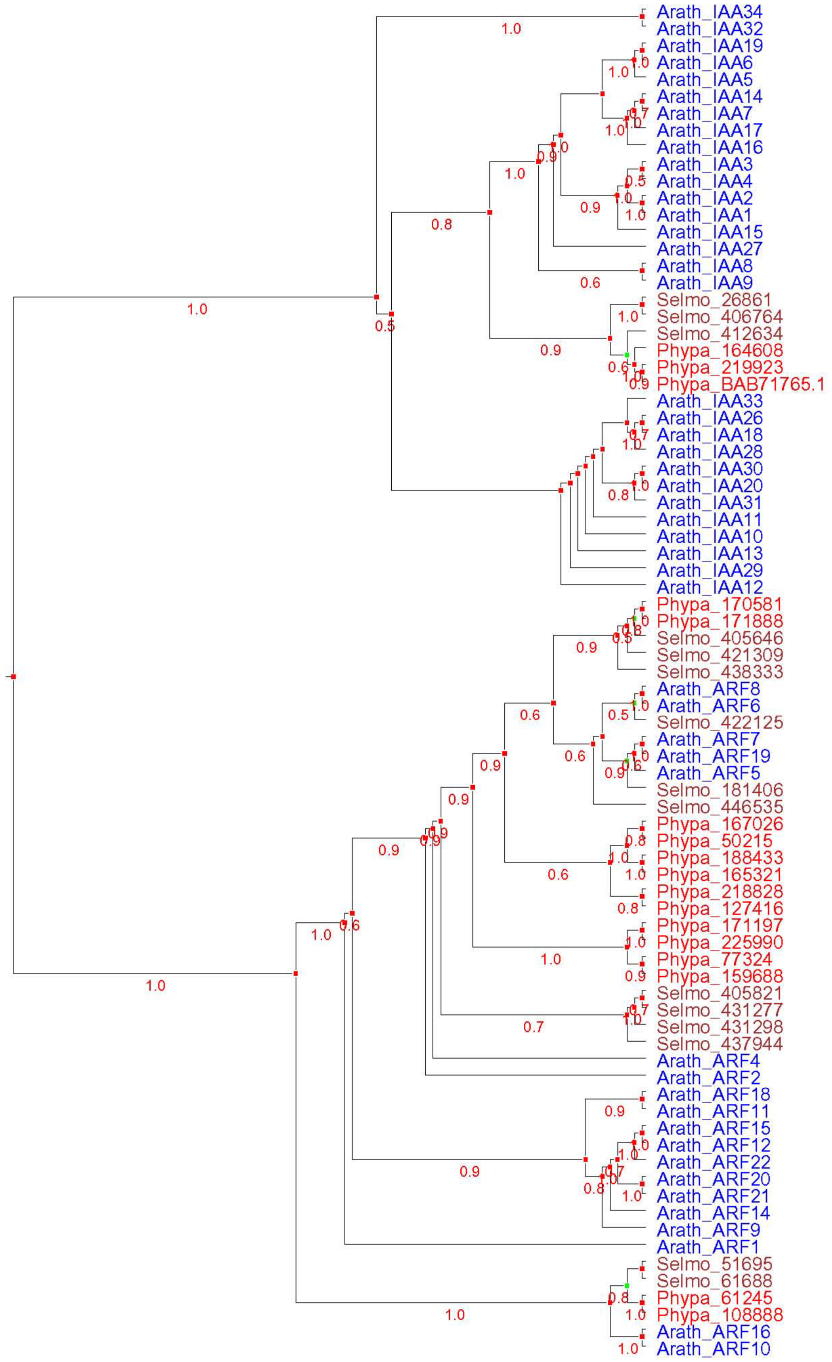

File 5. Phylogenetic relationship of *A. thaliana*, *S. moellendorffii* and *P. patens* ARF and Aux/IAA proteins (Bayesian inference) was reconciled with Notung using the species tree (Phypa, (Selmo, Arath)).
