# Supplementary material for: The evolution of nuclear auxin signalling
Source: BMC Evol Biol. 2009 Jun 3;9:126. doi: 10.1186/1471-2148-9-126 (PMC2708152; doi:10.1186/1471-2148-9-126)
Supplement: Additional file 6 — Phylogeny of A. thaliana and P. trichocarpa Aux/IAA (A) and ARF (B) proteins. Boxes identify nodes tested for positive selection. [file 1471-2148-9-126-S6.pdf]

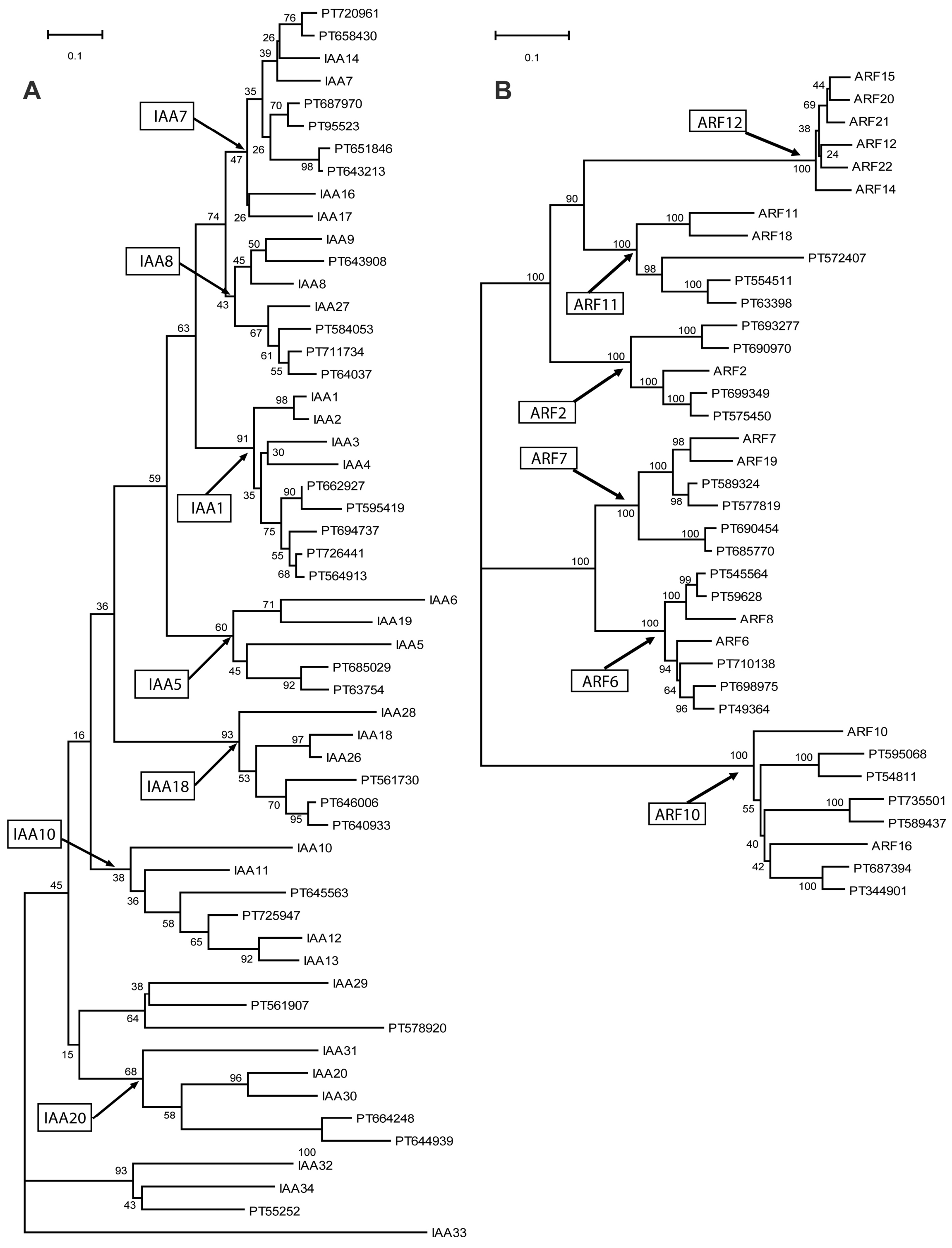

File 6. Phylogeny of *A. thaliana* and *P. trichocarpa* Aux/IAA (A) and ARF (B) proteins. Boxes identify nodes tested for positive selection.
