# Supplementary figures and images for: The evolution of nuclear auxin signalling
Source: BMC Evol Biol. 2009 Jun 3;9:126. doi: 10.1186/1471-2148-9-126 (PMC2708152; doi:10.1186/1471-2148-9-126)

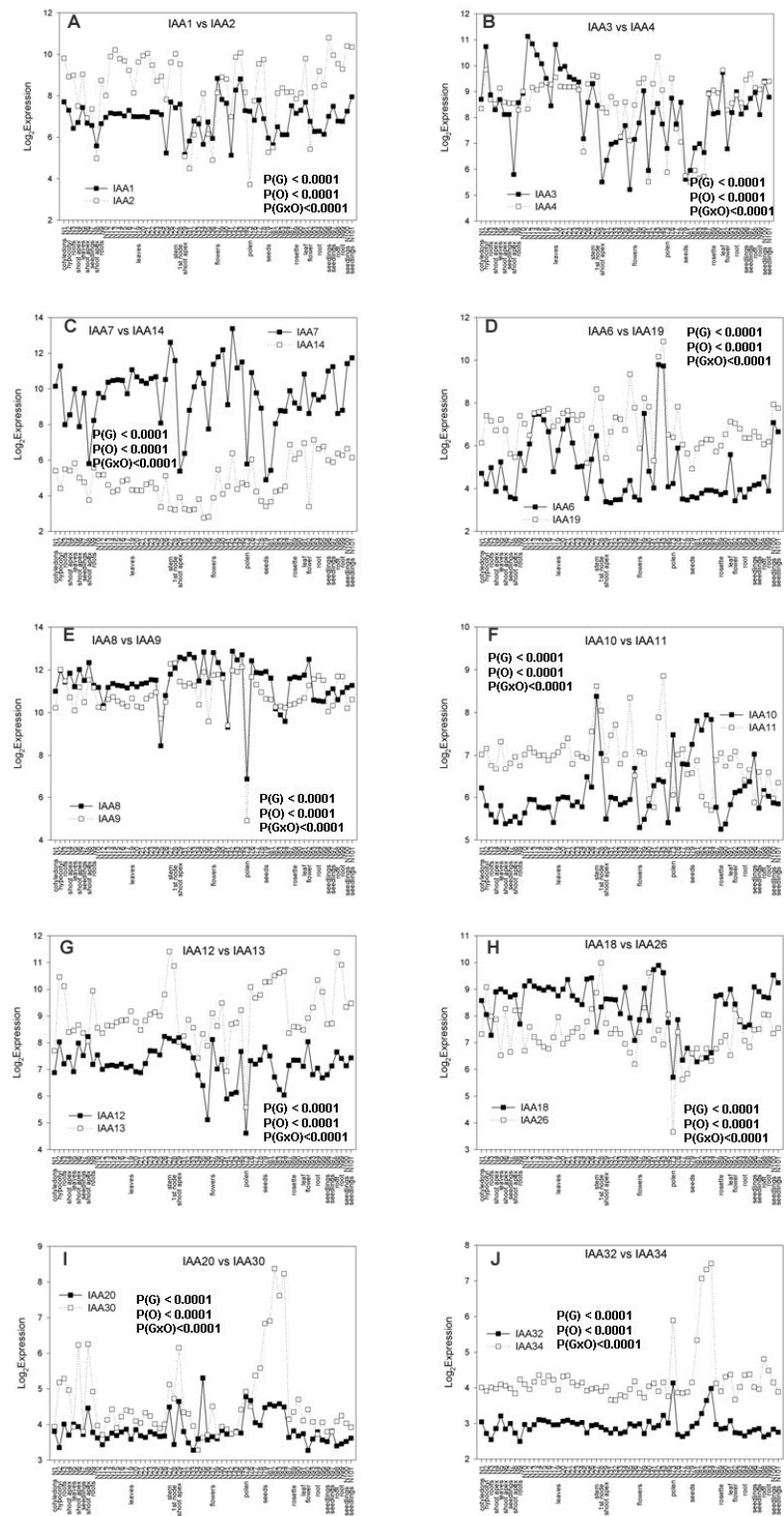

File 7. Expression pattern of paralogous pairs of *A. thaliana* Aux/IAA genes (A-J).

Supplement: Additional file 7 — Expression pattern of paralogous pairs of A. thaliana Aux/IAA genes (A-J). gcRMA normalized data were used. Three biological replications were used to generate the data set. The two-way ANOVA was used to partition the gene (G), sample (S) and GxS interaction effects. [file 1471-2148-9-126-S7.pdf]
