# Supplementary material for: The evolution of nuclear auxin signalling
Source: BMC Evol Biol. 2009 Jun 3;9:126. doi: 10.1186/1471-2148-9-126 (PMC2708152; doi:10.1186/1471-2148-9-126)
Supplement: Additional file 8 — Phylogenetic relationship of A. thaliana, S. moellendorffii and P. patens ARF proteins. Reconciled tree based on Bayesian inference. Length of middle region was normalized and transformed into a continuous character matrix. [file 1471-2148-9-126-S8.pdf]

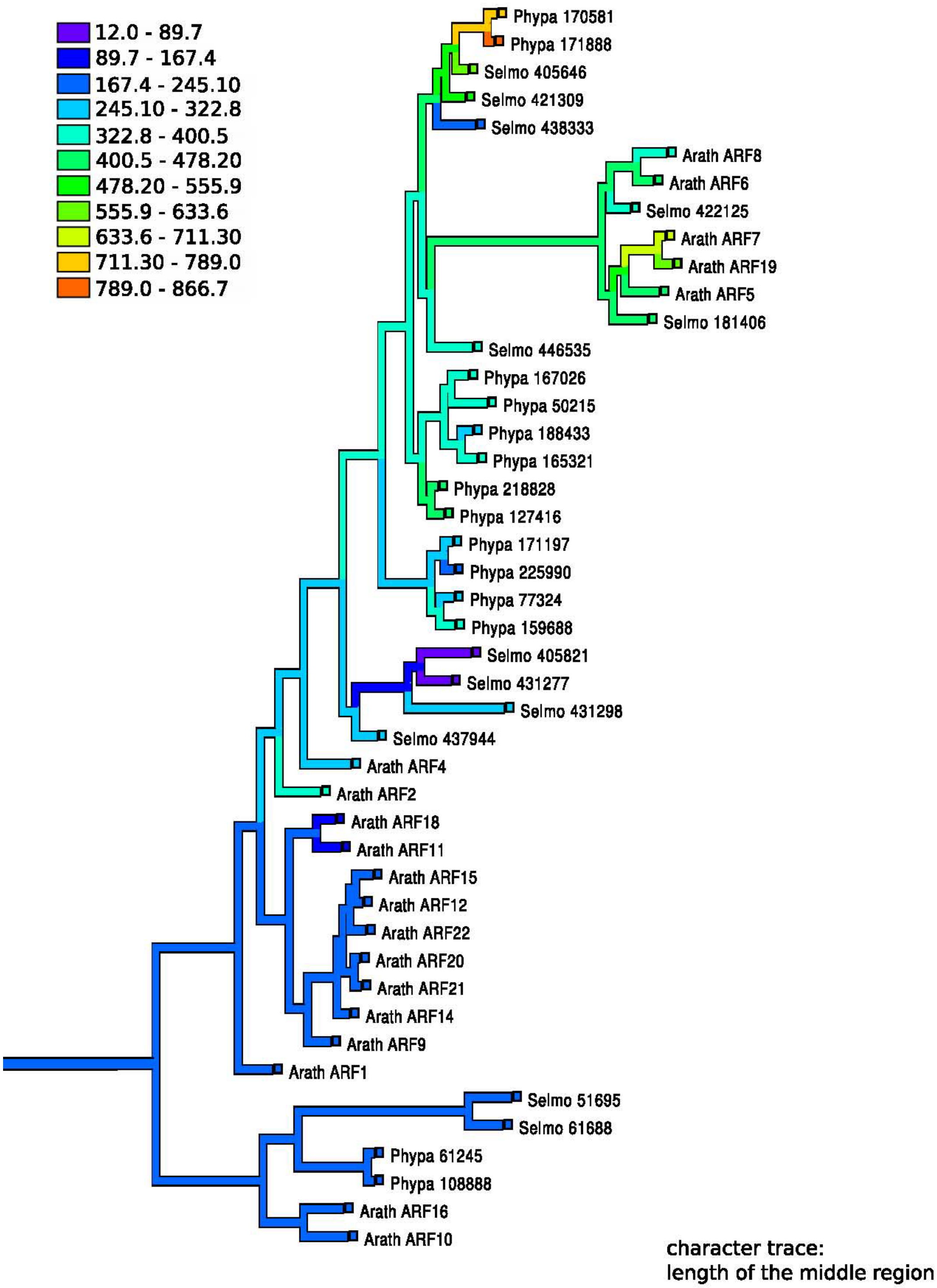

File 8. Phylogenetic relationship of *A. thaliana*, *S. moellendorffii* and *P. patens* ARF proteins (reconciled tree based on Bayesian inference). Length of middle region was normalized and transformed into a continuous character matrix.
