# Supplementary material for: The evolution of nuclear auxin signalling
Source: BMC Evol Biol. 2009 Jun 3;9:126. doi: 10.1186/1471-2148-9-126 (PMC2708152; doi:10.1186/1471-2148-9-126)
Supplement: Additional file 9 — Detailed comparison of A. thaliana, P. patens and S. moellendorffii ARFs. Here we present details of the middle region of ARFs, the presence of domain III and IV, amino acid frequency for Q, S, G, P, L, M, the total length of proteins, and the presence of amino acid-rich domains using ScanProsite. [file 1471-2148-9-126-S9.pdf]

Table S1. Detailed comparison of *A.thaliana* , *P.patens* and S.moellendorffii ARFs

Proteins	MR		length	III	IV	% by frequency						length	ScanProsite
	start	end				Q	S	G	P	L	M		
ARF1	355	543	189	+	+	5.8	16.4	7.9	12.7	3.7	0.0	665	P-rich
ARF2	395	734	340	+	+	5.9	12.7	5.9	8.2	6.8	2.4	859	
ARF3	390	566	177	-	-	5.1	14.1	13	6.2	5.7	0.0	608	
ARF4	407	666	260	+	+	5.4	11.9	8.5	10	6.9	2.3	788	
ARF5	392	798	407	+	+	10.1	15.1	5.4	6.7	7.9	4.4	902	M-rich
ARF6	359	795	437	+	+	17.2	16	6.6	7.8	10.1	4.1	933	Q-rich
ARF7	359	1039	681	+	+	22.8	12.3	6.5	7.5	11	2.4	1165	Q-/S-rich
ARF8	358	706	349	+	+	14.3	11.5	8.6	8	11.8	3.7	811	Q-rich
ARF9	344	524	181	+	+	6.1	17.7	1.7	7.2	5	2.8	638	
ARF10	383	581	199	+	+	6	11.1	8	7.5	8.5	3.0	693	
ARF11	352	491	140	+	+	7.9	22.9	2.1	8.6	5.7	0.0	601	
ARF12	354	512	159	+	+	5	10.7	4.4	8.2	10.7	4.4	593	
ARF13	354	463	110	-	-	5.6	13.9	3.7	5.6	13.9	1.9	505	
ARF14	354	511	158	+	+	7	13.9	3.8	7	8.9	3.2	605	
ARF15	359	517	159	+	+	6.9	12	5	8.2	10.1	3.8	598	
ARF16	385	585	201	+	+	3.5	14.9	7.5	11.5	10	1.0	670	
ARF17	379	506	128	-	-	4.9	12.3	11.5	14.8	8.2	3.3	585	
ARF18	356	490	135	+	+	8.9	16.3	3	15.6	5.9	1.5	602	
ARF19	357	959	603	+	+	18.9	12.9	5.6	8.1	7.3	4.2	1086	Q-rich
ARF20	352	496	145	+	+	6.2	10.3	4.8	6.2	10.3	4.1	590	
ARF21	354	512	159	+	+	6.9	12	4.4	8.2	10.7	3.8	606	
ARF22	352	510	159	+	+	6.3	12	5	8.2	10.7	3.8	598	
ARF23				-	-							222	
all_Phypa_167026	537	890	354	+	+	9.6	11.3	7.3	9	11.6	5.1	1019	
all_Phypa_218828	263	648	386	+	+	7.8	15.3	7.3	8	7	5.3	782	
all_Phypa_165321	362	727	366	+	+	8.2	12.8	9	11.8	9	3.8	849	
all_Phypa_188433	373	664	292	+	+	8.2	13	7.9	10.6	8.2	2.7	786	
all_Phypa_159688	498	835	338	+	+	4.4	15.1	5.6	11.2	8.6	0.9	945	
all_Phypa_77324	651	949	299	+	+	4.7	15.1	5	11.2	9.5	0.8	1059	
all_Phypa_225990	338	539	202	+	+	4.5	14.4	6.4	12.4	8.4	1.0	642	
all_Phypa_171197	742	1026	285	+	+	4.9	11.9	4.9	11.6	9.5	2.1	1129	
all_Phypa_196920	424	639	216	-	-	7.4	12.5	6.5	10.7	9.7	1.4	715	
Phypa_50215	342	672	331	+	+	10	15.4	7	8.8	10.9	4.2	758	
Phypa_127416	348	742	395	+	+	8.9	15.2	8.6	8.9	7.1	4.6	876	
Phypa_108888	362	520	159	+	+	5.7	11.3	12	12	10.1	3.9	596	
Phypa_170581	70	717	648	+	+	14.5	13	9.3	7.7	7.7	3.9	825	Q-rich
all_Phypa_171888	180	788	609	+	+	14	12.3	9.4	9	7.7	4.8	896	Q-/P-rich
Selmo1_2_181406	351	773	423	+	+	28.6	9.9	3.3	6.9	9.2	2.6	908	Q-rich
Selmo1_2_437944	371	665	295	+	+	5.8	11.9	6.1	8.5	6.4	3.7	796	
Selmo1_2_61688	367	548	182	+	+	5	12.6	8.8	7.1	10.4	3.9	622	Q-rich
Selmo1_446535	353	663	311	+	+	11.9	10	5.1	13.8	8.7	4.2	825	Q-rich
Selmo1_422125	347	684	338	+	+	14.8	13.3	6.8	5.6	8.6	1.8	801	
Selmo1_2_81992	343	362	20	-	-							382	
Selmo1_2_51695	389	589	201	+	+	8	11.4	11.4	6	9.5	1.0	665	
Selmo1_421309	28	486	459	+	+	16.1	14.6	4.6	10.5	7.4	3.5	582	Q-/P-/H-rich
Selmo1_405646	19	560	542	+	+	9.8	13.7	10.9	7.6	7.2	3.5	643	Q-/G-/H-rich
