# Supplementary material for: The evolution of nuclear auxin signalling
Source: BMC Evol Biol. 2009 Jun 3;9:126. doi: 10.1186/1471-2148-9-126 (PMC2708152; doi:10.1186/1471-2148-9-126)
Supplement: Additional file 10 — Phylogenetic relationship of A. thaliana, S. moellendorffii and P. patens ARF proteins. Reconciled tree based on Bayesian inference. Q-rich regions are represented by the amino acid frequency normalized with the length of the MR. [file 1471-2148-9-126-S10.pdf]

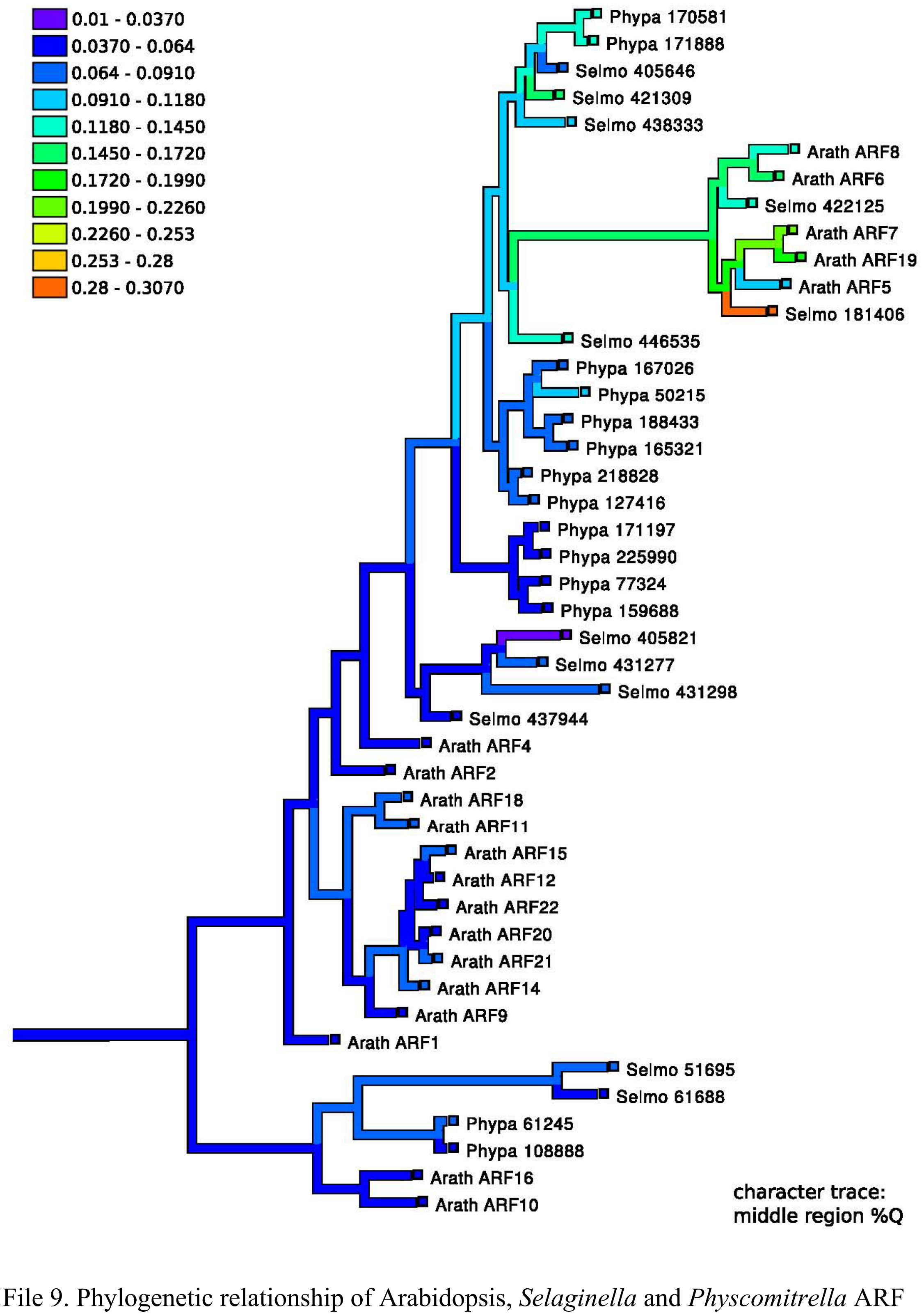

File 9. Phylogenetic relationship of Arabidopsis, *Selaginella* and *Physcomitrella* ARF proteins (reconciled tree based on Bayesian inference). Q-rich regions are represented by the amino acid frequency normalized with the length of the MR.
