# Supplementary material for: The evolution of nuclear auxin signalling
Source: BMC Evol Biol. 2009 Jun 3;9:126. doi: 10.1186/1471-2148-9-126 (PMC2708152; doi:10.1186/1471-2148-9-126)
Supplement: Additional file 11 — ARF protein sequence alignment of the middle regions in the ARF7 node of A. thaliana and P. trichocarpa. Arrows indicate sites at which positive selection was detected. Boxed amino acids indicate putative phosphorylation motifs. [file 1471-2148-9-126-S11.pdf]

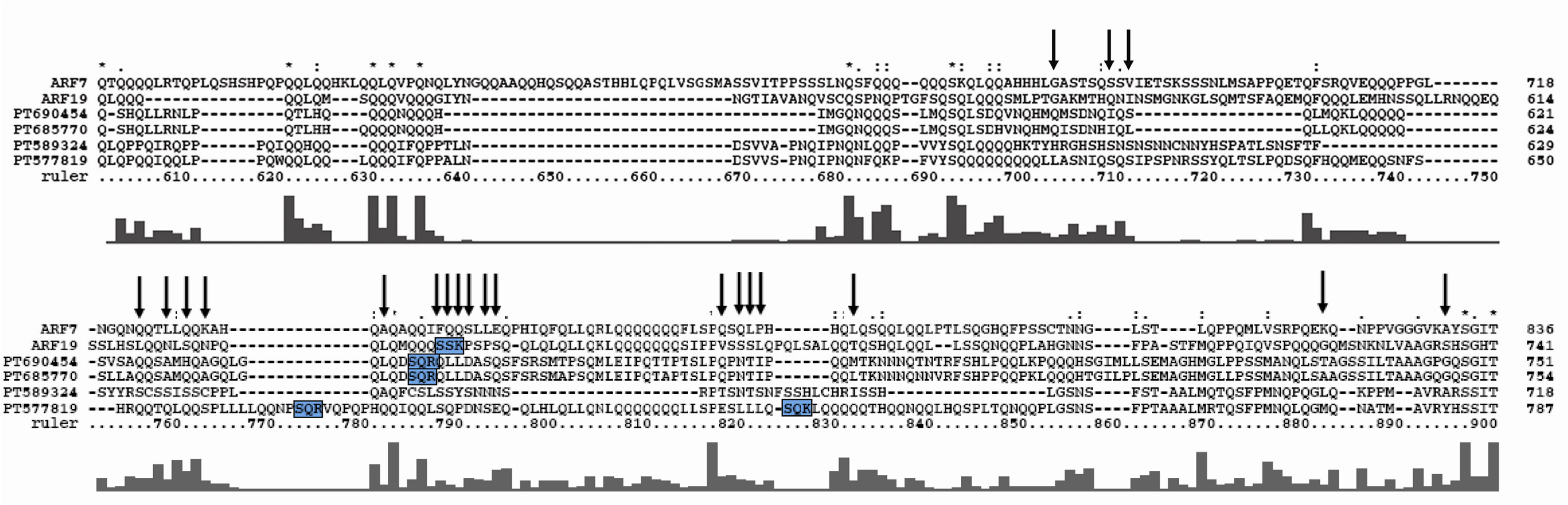

File 10. ARF protein sequence alignment of the middle regions in the ARF7 node of *A. thaliana* and *P. trichocarpa*. Arrows indicate sites at which positive selection was detected. Boxed amino acids indicate putative phosphorylation motifs.
