# Supplementary material for: The evolution of nuclear auxin signalling
Source: BMC Evol Biol. 2009 Jun 3;9:126. doi: 10.1186/1471-2148-9-126 (PMC2708152; doi:10.1186/1471-2148-9-126)
Supplement: Additional file 12 — Phylogenetic relationship (neighbor-joining (NJ) method) of A. thaliana, S. moellendorffii and P. patens GH3 proteins. PpGH3s are indicated in light blue. SmGH3s are indicated in light green. [file 1471-2148-9-126-S12.pdf]

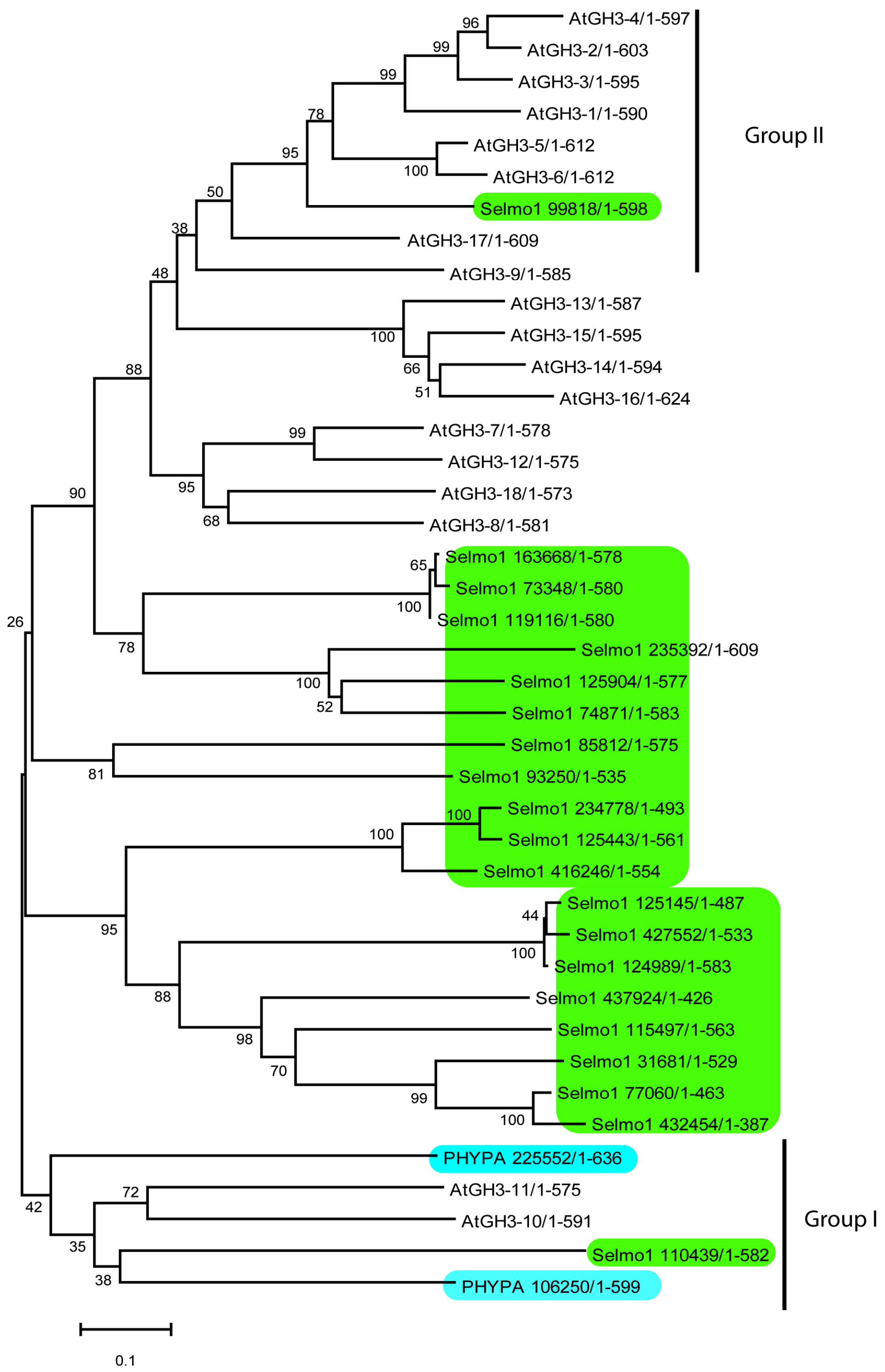

File 11. Phylogenetic relationship (neighbor-joining (NJ) method) of *A. thaliana, S. moellendorffii* and *P. patens* GH3 proteins.
