# Supplementary material for: The evolution of nuclear auxin signalling
Source: BMC Evol Biol. 2009 Jun 3;9:126. doi: 10.1186/1471-2148-9-126 (PMC2708152; doi:10.1186/1471-2148-9-126)
Supplement: Additional file 13 — Phylogenetic relationship (neighbor-joining (NJ) method) of A. thaliana and P. patens SAUR proteins. The P. patens SAURs are indicated in light blue.A. thaliana SAURs transcriptionally up-regulated by auxin are indicated in purple. [file 1471-2148-9-126-S13.pdf]

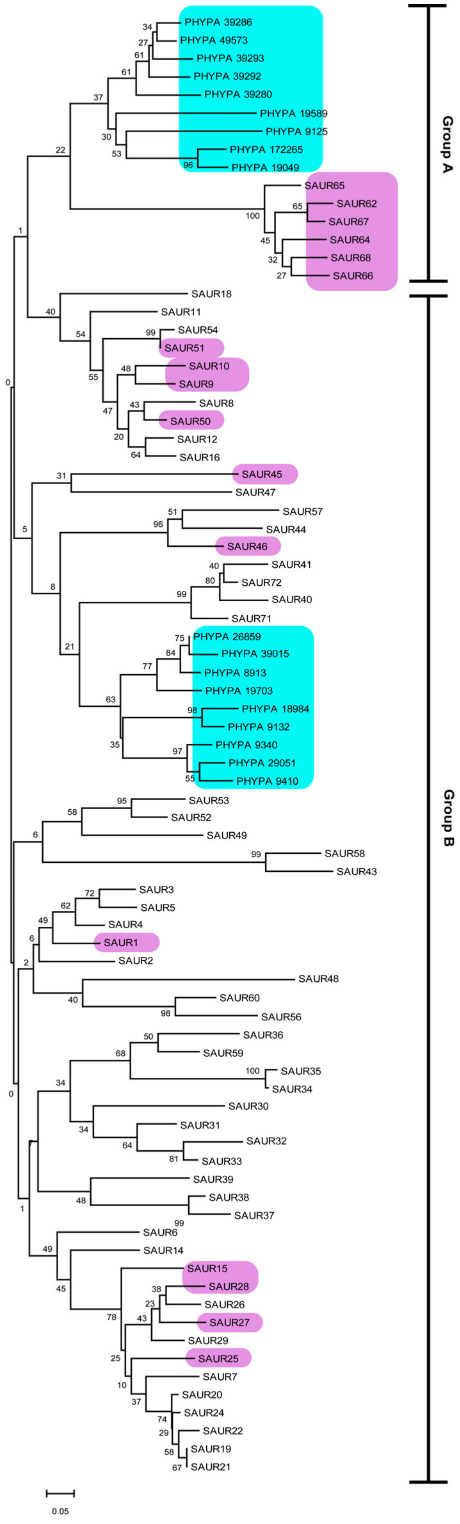

File 12. Phylogenetic relationship (neighbor-joining (NJ) method) of *A. thaliana* and *P. patens* SAUR proteins (light blue). Arabidopsis SAURs transcriptionally up-regulated by auxin are indicated in purple.
