# Supplementary material for: The evolution of nuclear auxin signalling
Source: BMC Evol Biol. 2009 Jun 3;9:126. doi: 10.1186/1471-2148-9-126 (PMC2708152; doi:10.1186/1471-2148-9-126)
Supplement: Additional file 14 — Phylogenetic relationship (neighbor-joining (NJ) method) of A. thaliana and P. patens LBD proteins. LBD proteins of P. patens are indicated in light green. A. thaliana LBDs transcriptionally up-regulated by auxin are indicated in purple. [file 1471-2148-9-126-S14.pdf]

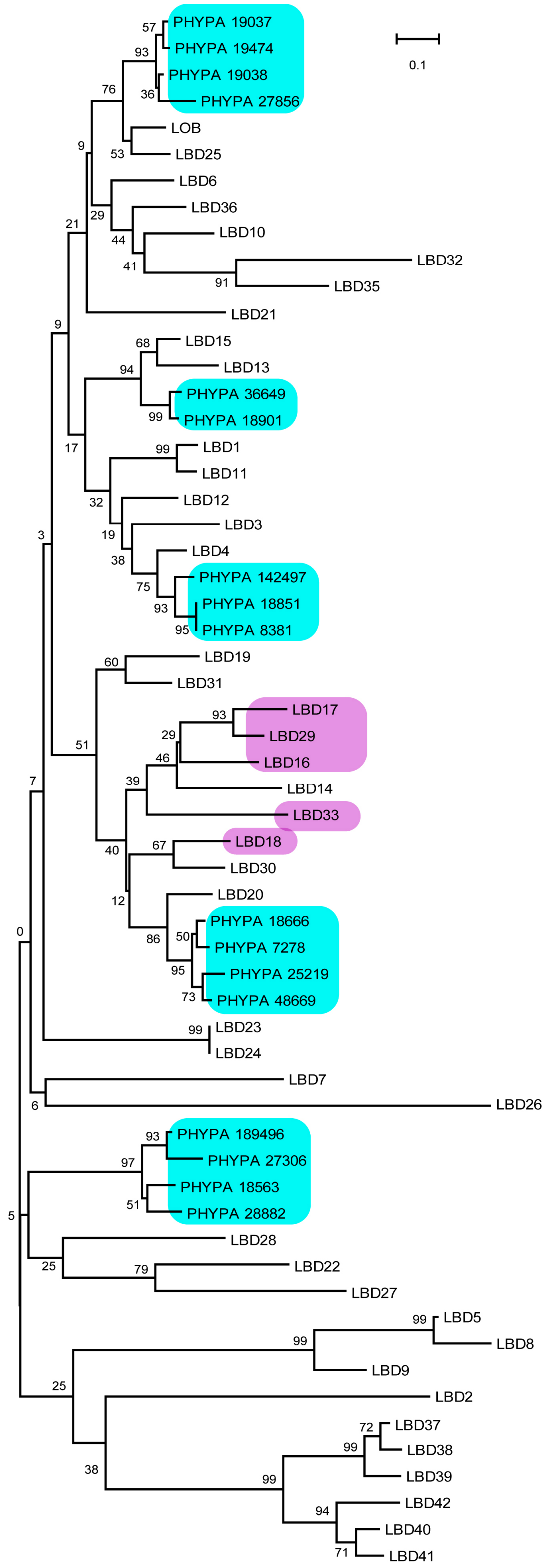

File 13. Phylogenetic relationship (neighbor-joining (NJ) method) of *A. thaliana* and *P. patens* LBD proteins.
